# In Cerebellar Atrophy of 12-Month-Old ATM-Null Mice, Transcriptome Upregulations Concern Most Neurotransmission and Neuropeptide Pathways, While Downregulations Affect Prominently Itpr1, Usp2 and Non-Coding RNA

**DOI:** 10.3390/cells12192399

**Published:** 2023-10-03

**Authors:** Marina Reichlmeir, Júlia Canet-Pons, Gabriele Koepf, Wasifa Nurieva, Ruth Pia Duecker, Claudia Doering, Kathryn Abell, Jana Key, Matthew P. Stokes, Stefan Zielen, Ralf Schubert, Zoltán Ivics, Georg Auburger

**Affiliations:** 1 Goethe University Frankfurt, University Hospital, Clinic of Neurology, Exp. Neurology, Heinrich Hoffmann Str. 7, 60590 Frankfurt am Main, Germany; marina.reichlmeir@kgu.de (M.R.); jcanetpons@gmail.com (J.C.-P.); jana.key@kgu.de (J.K.); 2 Transposition and Genome Engineering, Research Centre of the Division of Hematology, Gene and Cell Therapy, Paul Ehrlich Institute, 63225 Langen, Germany; wasifa.nurieva@pei.de (W.N.); zoltan.ivics@pei.de (Z.I.); 3 Division of Pediatrics, Pulmonology, Allergology, Infectious Diseases and Gastroenterology, Children’s Hospital, University Hospital, Goethe-University, 60590 Frankfurt am Main, Germany; ruthpia.duecker@kgu.de (R.P.D.); szielen@t-online.de (S.Z.); ralf.schubert@kgu.de (R.S.); 4 Dr. Senckenberg Institute of Pathology, University Hospital Frankfurt, 60590 Frankfurt am Main, Germany; c.doering@em.uni-frankfurt.de; 5 Cell Signaling Technology, Inc., Danvers, MA 01923, USA; kabell@cellsignal.com (K.A.); mstokes@cellsignal.com (M.P.S.); 6 Respiratory Research Institute, Medaimun GmbH, 60596 Frankfurt am Main, Germany

**Keywords:** cerebellar ataxia, cytoplasmic ATM, synaptic pathology

## Abstract

The autosomal recessive disorder Ataxia-Telangiectasia is caused by a dysfunction of the stress response protein, ATM. In the nucleus of proliferating cells, ATM senses DNA double-strand breaks and coordinates their repair. This role explains T-cell dysfunction and tumour risk. However, it remains unclear whether this function is relevant for postmitotic neurons and underlies cerebellar atrophy, since ATM is cytoplasmic in postmitotic neurons. Here, we used ATM-null mice that survived early immune deficits via bone-marrow transplantation, and that reached initial neurodegeneration stages at 12 months of age. Global cerebellar transcriptomics demonstrated that ATM depletion triggered upregulations in most neurotransmission and neuropeptide systems. Downregulated transcripts were found for the ATM interactome component *Usp2*, many non-coding RNAs, ataxia genes *Itpr1*, *Grid2*, immediate early genes and immunity factors. Allelic splice changes affected prominently the neuropeptide machinery, e.g., *Oprm1*. Validation experiments with stressors were performed in human neuroblastoma cells, where ATM was localised only to cytoplasm, similar to the brain. Effect confirmation in SH-SY5Y cells occurred after ATM depletion and osmotic stress better than nutrient/oxidative stress, but not after ATM kinase inhibition or DNA stressor bleomycin. Overall, we provide pioneer observations from a faithful A-T mouse model, which suggest general changes in synaptic and dense-core vesicle stress adaptation.

## 1. Introduction

The disease Ataxia Telangiectasia (A-T) is autosomal recessively inherited, shows a prevalence of 1:100,000 inhabitants, manifests in childhood and shortens lifespan to 25 years on average [1,2,3]. The diagnostic initial signs include problems of balance (ataxia) and speech, together with uncontrolled eye movements, due to progressively impaired motor coordination in the cerebellar neural circuits, as well as a dilatation of capillary blood vessels (telangiectasia). Blood tests will reveal an abnormal elevation of the prenatal osmosis regulator AFP (alpha-fetoprotein), which should normally be downregulated in postnatal life to be substituted by albumin [4,5]. Recently, neurofilament light chain (NfL) has been described as a potential biomarker for neurodegeneration from the early stages of A-T [6,7]. In subsequent years, a combined immune deficiency will lead to infections of the sinus and lungs, and over time to bronchiectasis [8]. Among classical A-T patients, IgA deficiency correlates with the poorest prognosis [9]. Gonadal atrophy will ensue, with gametogenesis undergoing meiotic arrest in early prophase, due to abnormal synaptonemal complex assembly resulting in fragmented chromosomes [10,11]. Body weight and height decline with age, accompanied by deficient secretion of growth hormones (GH) and trophic factors such as blood IGF-1, suggesting age-associated nutrient regulation stress [12,13,14]. A-T patients are particularly vulnerable to ionising radiation and ultraviolet-B light (the UVB wavelength is responsible for sunburns on skin), so their risk of cancer is elevated, manifesting particularly lymphoma and leukaemia in childhood, and breast cancer in adulthood [15,16]. Among these disease phenotypes, only immune deficits, infertility, and cancer risks have been mechanistically explained by the crucial role of nuclear ATM (the protein kinase Ataxia Telangiectasia Mutated, where nonsense or missense mutations usually trigger the A-T phenotype) for the detection and repair of DNA double-strand breaks (DSB) [17]. These DNA damage responses (DDR) coordinated by ATM are required to generate adequate antibody diversity in rapidly proliferating lymphocytes via V(D)J and class switch recombination [18,19]. However, there is an ongoing debate (1) why the osmotic regulator AFP increases and blood vessels dilate, (2) why nutrients are inadequately controlled in growth, and (3) why selectively post-mitotic neurons in the cerebellum should undergo insidious atrophy [20,21,22,23]. 

More detailed insights about ATM cellular expression, its subcellular redistribution, its stable interaction partners and its transient phosphorylation targets, together with its downstream signalling effects, are urgently needed. Such knowledge would help to understand cerebellar pathogenesis and to design therapeutic approaches. Currently, we only know that cerebellar ATM is expressed mainly in excitatory glutamatergic granule neurons, but also in efferent inhibitory GABAergic Purkinje neurons [24], other cerebellar neurons and afferent neural projections from the brainstem (see https://mouse.brain-map.org/gene/show/11706 accessed on 1 October 2023), as well as glial and endothelial cells. Its expression levels change with stress/stimulus responses [25]. Immunohistochemical and ultrastructural evidence showed neuronal ATM to localise to the cytoplasmic more than the nuclear compartment [24,26]. In immunoblots of nuclear versus cytoplasmic protein extracts from mice at maximal age 6 weeks, cytoplasmic ATM was solidly detected in the cerebellum but not in the spleen or thymus, while nuclear ATM remained strongly predominant even in the cerebellum at this young age [27]. However, this might change in adult animals when neuronal circuitry and myelination are complete. Regarding the ATM interactome, it is important to note that ATM is a member of the PIKK family (phosphoinositide 3-kinase-related kinases), which is anchored at membranes via the FATC domain [28,29]. Most other PIKKs phosphorylate inositol lipids, while ATM and its homolog ATR were reported to target selectively serine or threonine followed by glutamine (SQ-TQ motif) amino acids within several hundred protein substrates identified so far [17]. ATM mutation affects the membrane interface between endoplasmic reticulum and mitochondria [30], as well as endosomes, peroxisomes, lysosomal and autophagic vesicles [24,31,32]. Upon endosomal association, ATM was found to interact with beta-Adaptin (AP1B1/AP2B1) and Neuronal Adaptin-like beta-subunit Protein (beta-NAP) [33]. The cytoplasmic portion of ATM prompted different studies about altered pathways there, and about additional ATM functions [34,35,36,37], but a conclusive mechanistic scenario has not emerged as yet. The association of ATM with presynaptic neurotransmitter-containing vesicles was also demonstrated [38], with a preferential binding to excitatory vesicles that contain VGLUT1 as a glutamate transporter to control their quantal size [39,40]. Pre- and post-synaptic swelling and loss of cytosolic texture were detectable by electron microscopy in ATM-null mouse cerebellar cortex already at age 2 months [41]. Cerebellar Purkinje pathology involves defects in calcium spike bursts and calcium currents, as well as the progressive reduction in spontaneous action potential firing frequency, from the age of 6 weeks to their maximal lifespan of 5 months in the absence of treatment [42]. Overall, the absence of ATM protein from its physiological membrane association in neuronal cytoplasm clearly triggers age-associated neurodegeneration, but it remains unclear to what degree ATM acts via its physical interactions with membrane lipids and proteins, versus its protein kinase activity. 

With regard to ATM presence as opposed to its kinase activity, it is important to know that mice expressing the kinase-deficient ATM exhibit an early embryonic lethality phenotype [43,44], whereas ATM-null mice are viable and their affection becomes apparent only for the immune system at early adult ages. This might suggest that the absence of ATM is sensed and mostly compensated for by cells, whereas substituting ATM function becomes much more difficult if it occupies the correct positions within its interactome, but fails to signal upon stress events. In mice, the ATM deficiency usually results in a shortened survival of haematopoietic cells, early frequent occurrence of lymphomas, and a lifespan over a few months only, so the manifestation of ataxia and cerebellar atrophy is usually prevented by an untimely death due to the immune deficit [41,45]. A dramatic extension of life expectancy from 4 to 12 months was achieved by bone-marrow transplants in ATM-null mice, and in such animals a decreased cerebellar size index was observed upon brain imaging at the age of 8 months [46]. 

Activation of normally inactive homodimeric ATM is differently regulated, when distinct stressors are applied. Variance in post-translational modifications and interaction partners of ATM exist. The DNA damage-dependent activation (e.g., by the DNA strand-breaking drug bleomycin, or ionizing radiation) involves Ser1981 autophosphorylation, Lys3016 acetylation by KAT5, interaction with the MRN protein complex (MRE11, RAD50 and NBS1) and ATM monomerisation [47,48,49]. In neuronal cells, strong excitation promotes immediate-early gene transcription via DNA-DSB, which are mediated by topoisomerase-1 cleavage complexes (TOP1cc), and have to be eliminated by ATM activation, otherwise toxic accumulation of R-loops will occur [50,51]. It is thought that ATM senses TOP1cc/R-loops and organises their removal, in a process that is impaired upon oxidative damage [17]. Indeed, elevated levels of R-loops were observed in ATM-null mouse testis, but not in brain tissue, at the age of 1 month [52]. Importantly, the R-loop activation of ATM promotes chromatin displacement of late-stage spliceosomes, so the alternative splicing in ATM mutants may be changed in genome-wide manner [53]. Some ATM-dependent changes in RNA processing were reported to be mediated by the nuclear splice regulator SAM68 [54]. Thus, RNA neurotoxicity via R-loops and SAM68, protein aggregation and unbalanced excitability have been proposed to underlie the ataxia and cerebellar atrophy in A-T, in view of similar clinico-pathological findings in other monogenic spinocerebellar ataxias where mutant AOA2, FRDA, ATXN2, ITPR1 trigger similar cytosolic pathways in pathogenesis [17,55,56,57]. However, few other data are available to judge the overlap in pathogenesis between diverse monogenic ataxias, and to decide which other cerebellar ataxias are closest to A-T. 

In contrast to these mechanisms following DNA and RNA damage, the activation of ATM upon osmotic stress (e.g., by the drug chloroquine, or hypotonic shock) involves its interaction with ATMIN [47,58]. 

Furthermore, activation of nuclear ATM via nutrient deprivation (by 2-deoxyglucose exposure) is mediated by the inefficient assembly of a protein complex between the endoplasmic reticulum and mitochondrial membranes, which is composed by IP3R1 (gene symbol *ITPR1*), GRP75 (gene symbol *HSPA9*), and VDAC1. This inadequate assembly results in impaired release of Ca^2+^ and excitability in the human bronchial epithelial cell line HBEC3-KT [30]. 

Finally, the activation of ATM via oxidative stress (e.g., by the drug sodium arsenite, abbreviated as NaARS, or by hydrogen peroxide H_2_O_2_) involves Cys2991 disulfide bonds linking active ATM homodimers, but appears independent from the MRN complex [59]. Again, ATMIN plays a relevant role in the protection against oxidative stressors [60]. All these mechanistic insights were obtained in cell culture or in young adult animals. Thus, at present it remains completely unclear which of these stressors and molecular response mechanisms would play the prominent role in the cerebellum when the age-associated pathology manifests. 

For the present study, we analysed cerebellar homogenates from bone-marrow-transplanted, 12-month-old ATM-null mice, documenting their global transcriptome by oligonucleotide microarrays, in the hope of elucidating the impact of ATM for RNA-mediated stress responses. With this approach, we hoped to answer the following questions: (1) Which impact exists on transcript levels of known ATM interactors, or known ATM phosphorylation substrates? (2) Which dysregulations occur in phosphoinositide pathway membrane factors, in vesicular factors, or in calcium homeostasis factors? (3) To what degree is the altered neuronal excitability reflected by dysregulations of neurotransmission factors or immediate early genes? (4) Were some dysregulations already observed in telangiectasia, in general growth deficit, or selective cerebellar atrophy, e.g., as known disease genes of other cerebellar ataxias? Such findings would define the mechanistic overlap with other genetic disorders. (5) Are there strong dysregulations of novel character outside these already explored pathways? 

Validation work in vitro with further methods and samples was performed to answer additional questions: (6) Whether ATM in the adult cerebellum is still mostly nuclear with solid cytosolic presence, and if the human neural SH-SY5Y cell line is a good model of ATM distribution, was assessed with differential detergent fractionation. (7) To understand if ATM kinase activity or ATM protein presence triggers such dysregulation events, we exposed the human neuroblastoma cell line SH-SY5Y either to the ATM kinase inhibitor drug KU-55933, or to stable ATM knockdown (KD) via shRNA, and quantified transcript alterations with RT-qPCR. (8) To identify which specific stressor agents provide the best model for the age-effect on dysregulated cerebellar transcripts in ATM-null mice, human SH-SY5Y cells with *ATM*-KD were assessed with RT-qPCR and quantitative immunoblots. 

Overall, in ATM-null mice at advanced age, several strong cerebellar mRNA dysregulations were documented, and their reproducibility in cell culture after ATM depletion and stressor administration provided criteria to distinguish primary from secondary effects.

## 2. Materials and Methods

### 2.1. Animal Model of Ataxia-Telangiectasia

To study the cerebellar atrophy of A-T, we used ATM-null mice (strain 002,753 from the Jackson depository, also denominated as Atm^tm1Awb/F^ or ATM-null or *Atm*^−/−^) [45] in the 129/SvEv genetic background. Animal procedures were approved by the regional authority (RPDA number FK/1034 with date of approval 17 March 2015). Mice were housed in accordance with the German Animal Welfare Act, Council Directive of 24 November 1986 (86/609/EWG) Annex II, ETS123, and the EU Directive 2010/63/EU, at the FELASA-certified Central Animal Facility (ZFE) of Frankfurt University Medical School, employing type II L cages (365 × 207 × 140 mm^3^, floor area 530 cm^2^), with mutants and wildtype (WT) controls being bred and aged in parallel, under controlled conditions of temperature, humidity, and light/dark cycles of 12 h, providing food and water ad libitum. Genotyping of ear-punch DNA was carried out using PCR procedures as described previously [61].

### 2.2. Intravenous Transplantation of Whole Bone Marrow Cells

As a conditioning regimen, the recipient mice received 0.125 mg/mL anti-CD4 antibody (clone GK1.5, Sigma, Steinheim, Germany) and 0.125 mg/mL anti-CD8 antibody (clone 53–6.7, Sigma) 7 days before bone marrow transplantation (BMT), and then a second dose of each antibody together with 200 mg/kg cyclophosphamide (80 mg/mL, Sigma-Aldrich, St. Louis, MO, USA) 1 day before BMT for nonmyeloablative conditioning. Bone marrow cells were harvested in a sterile manner from CD-90.2 depleted, *ATM*-competent donor animals on the day of BMT, and 5 × 10^6^ bone marrow cells were injected intravenously into conditioned recipients [46,62]. Ageing of mutants and sex-/age-matched WT animals until 12 months was closely monitored after the intervention, continuously assuring that lymphoma and immunological deficits were not threatening the mice. Dissection of four ATM-null versus four matched WT mice occurred after cervical dislocation, snap-freezing the fresh cerebellar tissue in liquid nitrogen for oligonucleotide microarray surveys and subsequent validation experiments by RT-qPCR.

### 2.3. Global Transcriptome Survey

Total RNA was extracted from frozen tissue using TRIzol reagent (Sigma-Aldrich, St. Louis, MO, USA), according to the manufacturer’s instructions. The RNA integrity number (RIN) was assessed using a 2100 Bioanalyzer RNA 6000 Nano Assay (Agilent Technologies, Santa Clara, CA, USA) and its concentration determined with NanoDrop Spectrophotometer (Thermo Fisher Scientific, Waltham, MA, USA). Samples were kept at −80 °C until use. Then, 1 μg of RNA was pre-treated with DNase amplification grade (Invitrogen, Carlsbad, CA, USA). The Gene Chip^TM^ WT PLUS Reagent Kit (Applied Biosystems, Waltham, MA, USA) was used to generate single-stranded cDNA (ss-cDNA), which was fragmented and labelled right before hybridisation to Clariom D arrays (Thermo Fisher Scientific, Waltham, MA, USA). The signals were documented with the Affymetrix Gene Chip Scanner, and data were processed with the Transcriptome Analysis Console (TAC) 4.0.1 (Applied Biosystems, Waltham, MA, USA) software using default algorithm parameters. The complete gene expression data set was deposited publicly in the Gene Expression Omnibus under accession number GSE241955.

### 2.4. Bioinformatics Analysis of Global Transcriptome Data

The distribution of all microarray oligonucleotides that showed differential dysregulation with actual significance (false discovery rate FDR *p*-value < 0.05) in cerebella of 12-month-old *Atm*-deficient mouse cerebella were displayed as a volcano plot in Figure 2a (a logarithmic display where log2 values of fold change make downregulations in a green colour and upregulations in a red colour easily comparable on the *X*-axis, and −log10 of FDR *p*-values on the *Y*-axis enables graphic representation of outliers). The absolute numbers and percentages of downregulations and upregulations with nominal significance (gene level *p*-value < 0.05, fold change >1.2 or <−1.2) across the transcriptome, and the overrepresentation of Non-Coding transcripts among downregulations, versus overrepresentation of coding and Multiple-Complex transcripts among upregulations, are displayed as pie charts in Figure 2b. In the Clariom D microarray, there are nine predefined oligonucleotide groups: Non-Coding, Multiple Complex (containing more than one of the other groups), Coding, Pseudogene, Precursor microRNA, small RNA, Ribosomal, Unassigned, and tRNA. All transcript dysregulations with nominal significance were subjected to Gene Ontology (GO)-enrichment analysis via PANTHER (http://geneontology.org/, accessed on 24 January 2023). Fisher’s Exact was used for statistical evaluation, and correction was done by FDR. PANTHER Overrepresentation Test was carried out separately for upregulations (Figure 2d) and downregulations (Figure 2c), in each case calculating the enrichment for GO biological process (upper panel) and GO molecular function (lower panel). The resulting GO terms were sorted by Fold Enrichment, and the top 10 hits are displayed as bar graphs. Given that the Clariom D microarrays represent practically each exon of all coding transcripts, further analyses of alternative splicing were possible at genome-wide level (Figure 5). As filtering criteria, genes with exon splicing index >5 or <−5, and significance with FDR *p*-value < 0.25 were selected (Figure 5a). Among these, pathway enrichment studies using the STRING (Search Tool for the Retrieval of Interacting Genes/Proteins) webplatform (https://string-db.org/, last accessed on 16 June 2021) demonstrated an overrepresentation for the terms “Neuropeptide signaling pathway”, “Regulation of neurotransmitter levels” and “Synapse organization” (shown as an interaction plot in Figure 5b).

### 2.5. Neuroblastoma Cell Culture and Treatments

Parental SH-SY5Y human neuroblastoma cell line was cultured in high glucose DMEM (Thermo Fisher Scientific, Waltham, MA, USA, 21969-035) supplemented with 10% FCS (Thermo Fisher Scientific, Waltham, MA, USA, A3160802), 1% L-Glutamine (Thermo Fisher Scientific, Waltham, MA, USA, 25030-024) and 0.1% Penicillin/Streptomycin (Thermo Fisher Scientific, Waltham, MA, USA, 15140-122). *ATM* knockdown SH-SY5Y cells were kept in a selection medium, as explained later. 

Stable knockdown of *ATM* in SH-SY5Y was achieved via lentiviral transduction of five different MISSION short hairpin RNAs targeting *ATM* (shRNA, commercially available at Sigma-Aldrich, St. Louis, MO, USA) and one non-targeting control shRNA, targeting no known mammalian genes (Sigma-Aldrich, St. Louis, MO, USA, SHC002, hereafter referred to as NT CTRL, gift from Prof. Dr. Donat Kögel) in mammalian expression vector pLKO.1. The shATM sequences were:

sh*ATM*#1–5′CCGGCCAAGGTCTATGATATGCTTACTCGAGTAAGCATATCATAGACCTTGGTTTTTTG-3′ (cat.no. TRCN0000194861),
sh*ATM*#2–5′CCGGTGGTCAAATACTTCATCAAATCTCGAGATTTGATGAAGTATTTGACCATTTTTG-3′ (cat.no. TRCN0000245108),
sh*ATM*#3–5′CCGGTGATGGTCTTAAGGAACATCTCTCGAGAGATGTTCCTTAAGACCATCATTTTTG-3′ (cat.no. TRCN0000010299),
sh*ATM*#4–5′CCGGCCTTTCATTCAGCCTTTAGAACTCGAGTTCTAAAGGCTGAATGAAAGGTTTTTG-3′ (cat.no TRCN0000039948),
sh*ATM*#5–5′CCGGGCCTCCAATTCTTCACAGTAACTCGAGTTACTGTGAAGAATTGGAGGCTTTTTG-3′ (cat.no. TRCN0000039951).

Stable KD cells were generated by transfecting 2 µg of the respective shRNA or NT CTRL plasmid DNA, 1.5 µg gag/pol plasmid DNA (psPAX2, Addgene #12260) and 0.5 µg VSV-G envelope plasmid DNA (pMD2.G, Addgene #12259) into HEK293T cells using FuGENE HD transfection reagent (Promega, Fitchburg, WI, USA, E2311) following the manufacturer’s instructions. psPAX2 was a gift from Didier Trono (Addgene plasmid # 12260; http://n2t.net/addgene:12260, accessed on 1 October 2023; RRID:Addgene_12260). pMD2.G was a gift from Didier Trono (Addgene plasmid # 12259; http://n2t.net/addgene:12259, accessed on 1 October 2023; RRID:Addgene_12259). After 16 h and 40 h post-transfection, the viral supernatant was collected, pooled, sterile-filtered (0.45 µm) and applied to the SH-SY5Y cells in a 1:1 mixture with fresh medium supplemented with 3 µg/mL polybrene (Sigma-Aldrich, St. Louis, MO, USA, TR-1003). SH-SY5Y cells were transduced for 24 h and selected via bulk selection using puromycin (Santa Cruz Biotechnology, Dallas, TX, USA, sc-108071). To achieve this, the SH-SY5Y culture medium was supplemented with 1.25 µg/mL puromycin as determined by the kill curve in parental cells. Cells were generally maintained in puromycin selection medium in order to reduce the probability of KD loss. 

After expansion, sh*ATM*-containing cells were assessed for protein and RNA levels, and sh*ATM*#2 was selected for further experiments after achieving the best KD. 

For stress experiments, parental and knockdown cells were treated with chloroquine (CQ, Sigma-Aldrich, St. Louis, MO, USA, C6628) for osmotic stress, bleomycin (BLEO, Merck Millipore, Burlington, MA, USA, 203408-250MG) for genotoxic stress, sodium arsenite (NaARS, Sigma-Aldrich, St. Louis, MO, USA, S7400-100G) for oxidative stress, and LY-294002 (LY, Cayman Chemical Company, Ann Arbor, MI, USA, 70920) for trophic stress via phosphoinositide 3-kinase (PI3K) inhibition. An amount of 20 µM chloroquine was administered for 24 h with sterile water as a control. BLEO treatment was at 5 µM for 8 h, with DMSO as the control condition. NaARS was delivered at 0.5 mM for 45 min, water serving as a control. LY was administered at a concentration of 10 µg/mL for 24 h, with DMSO as a control. For pre-treatment of parental SH-SY5Y cells with the ATM inhibitor KU-55933 (KU, Selleckchem, Houston, TX, USA, S1092), 10 µM were used over 30 min, prior to the cell stress exposure, with DMSO as a control. 

Cells were harvested in Phosphate Buffered Saline (PBS) using cell scrapers. After centrifugation, pellets were frozen until usage in either nucleic acid analysis via RT-qPCR, or protein analysis via immunoblotting or subcellular fractionation.

### 2.6. Reverse Transcriptase Real-Time Quantitative Polymerase Chain Reaction (RT-qPCR)

Total RNA was isolated from either the mouse cerebellum or cell pellets. RNA extraction was performed using TRI reagent (Sigma-Aldrich, St. Louis, MO, USA) following the manufacturer’s protocol. To generate cDNA from RNA samples, the SuperScript IV Kit (Invitrogen, Carlsbad, CA, USA) was used. A total amount of 1 μg RNA was first digested with ezDNase enzyme (Invitrogen, Carlsbad, CA, USA) for purification and finally reverse transcribed following the manufacturer’s instructions. For gene expression analysis, RT-qPCR was performed using TaqMan Gene Expression Assays^TM^ (Thermo Fisher Scientific, Waltham, MA, USA). For this purpose, cDNA from 10 ng total RNA was used with 2× FastStart Universal Probe Master ROX (Roche, Basel, Switzerland) and the corresponding TaqMan Assay. The reaction was performed in a StepOnePlus Real-Time PCR Cycler (Applied Biosystems, Waltham, MA, USA). Data were analysed using the 2^−ΔΔCt^ method [63]. 

The following TaqMan Assays were used for murine transcripts:

*Atm*–Mm01177457_m1; *Atmin*–Mm01251229_m1; *Ecel1*–Mm00469610_m1;
*Foxo3*–Mm01185722_m1; *Grid2*–Mm00515053_m1; *Grin2b*–Mm00433820_m1;
*Grin2c*–Mm00439180_m1; *Grm4*–Mm01306128_m1; *Itpr1*–Mm00439907_m1;
*Mme*–Mm00485040_m1; *Nr4a1*–Mm01300401_m1; *Nr4a2*–Mm01278507_g1;
*Nr4a3*–Mm00450074_m1; *Oprm1* (Exon 2–3)–Mm01188089_m1;
*Oprm1* (Exon 5–6)–Mm01188387_m1; *Per1*–Mm00501813_m1;
*Rora*–Mm01173766_m1; *Slc17a6*–Mm00499876_m1; *Slc32a1*–Mm00494138_m1;
*Sst*–Mm00436671_m1; *Tac1*–Mm00436880_m1; *Tacr1*–Mm00436892_m1;
*Tbp*–Mm00446973_m1; *Usp2*–Mm00497452_m1.

The following TaqMan Assays were used for human transcripts:

*ATM*–Hs01112311_m1; *ATMIN*–Hs00739820_m1; *CAMK2A*–Hs00947041_m1;
*CAMK4*–Hs00174318_m1; *ECEL1*–Hs00191400_m1; *FOXO3*–Hs00818121_m1;
*GRID2*–Hs00910017_m1; *ITPR1*–Hs00976045_m1; *MME*–Hs01115452_m1;
*NR4A1*–Hs00374226_m1; *OPRM1*–Hs01053957_m1; *OPRM1* (Exon 1–2)–Hs01053956_m1;
*OPRM1* (Exon 3–4)–Hs00168570_m1; *PER1*–Hs00242988_m1; *RORA*–Hs00536545_m1;
*RRAGD*–Hs00222001_m1; *SGK1*–Hs00178612_m1; *TBP*–Hs9999910_m1;
*USP2*–Hs00275859_m1.

### 2.7. Immunoblotting

For protein analysis in the cerebellum, the tissues were lysed, homogenised in urea lysis buffer and sonicated on medium power (three 10 s bursts). Lysates were centrifuged at 18,000× *g* for 15 min. Protein content of the lysate was estimated using the Pierce 660 nM protein assay kit (Thermo Fisher Scientific, Waltham, MA, USA). Equal amounts of protein lysates (10 µg) were separated by SDS-Polyacrylamide gel electrophoresis (PAGE) (Bio-Rad, Hercules, CA, USA) and transferred to the nitrocellulose membrane (Merck Millipore, Burlington, MA, USA). Non-specific binding was blocked using 5% non-fat dry milk/TBS-T for 1 h at room temperature, and then the membrane was incubated with a primary antibody against ATM (#2873, Cell Signaling Technology, Danvers, MA, USA) or with β-Actin (ACTB, #4970, Cell Signaling Technology, Danvers, MA, USA) at 4 °C overnight in 5% BSA/TBS-T. The next day, membranes were washed with TBS-T (3 × 5 min each) and incubated with anti-rabbit IgG (H+L) (DyLight^TM^680 Conjugate) secondary antibody for 1 h. Antibody binding was visualised on the LI-COR Odyssey NIR (near infrared) imaging system.

For protein analysis in SH-SY5Y cells, samples were first lysed in RIPA buffer (50 mM TRIS/HCl pH 8.0, 150 mM NaCl, 1% NP-40, 0.5% sodium deoxycholate, 0.1% sodium dodecyl sulfate = SDS), containing HALT phosphatase inhibitors (Thermo Fisher Scientific, Waltham, MA, USA) and cOmplete proteinase inhibitors (Roche, Basel, CHE) for 30 min on ice. Following that, the lysates were briefly sonicated and subjected to Pierce BCA Protein Assay Kit (Thermo Fisher Scientific, Waltham, MA, USA) for determination of protein concentration following the manufacturer’s instructions. For the SDS-PAGE, 25 µg protein was used and denatured at 90 °C for 5 min. SDS-PAGE was carried out following standard procedures. Proteins were transferred on 0.2 µm nitrocellulose membranes (Bio-Rad, Hercules, CA, USA) and blocked in 5% bovine serum albumin (BSA, Carl Roth GmbH, Karlsruhe, Germany) in TBS-buffer containing 0.1% Tween-20 (Sigma-Aldrich, St. Louis, MO, USA) for 1 h. Primary antibodies were rabbit anti-ATM (Cell Signaling Technology, Danvers, MA, USA, #2873), mouse anti-pATM (S1981, Cell Signaling Technology, Danvers, MA, USA, #4526), mouse anti-α-tubulin (=TUBA, Sigma-Aldrich, St. Louis, MO, USA, T9026), mouse anti-GAPDH (Calbiochem, St. Louis, MO, USA, CB1001), mouse anti-vinculin (=VCL, Proteintech, Rosemont, IL, USA, 66305-1-Ig), mouse anti-HSP60 (Santa Cruz Biotechnology, Dallas, TX, USA, sc-13115), rabbit anti-LAMIN-A/C (=LAMIN, Abcam, Cambridge, UK, ab169532), rabbit anti-IP3 receptor (=ITPR1, Abcam, Cambridge, UK, ab5804), rabbit anti-PER1 (Proteintech, Rosemont, IL, USA, 13463-1-AP), rabbit anti-USP2 (Proteintech, Rosemont, IL, USA, 10392-1-AP). Incubation was performed overnight at 4 °C. Membranes were incubated with the respective secondary antibody IRDye 800CW goat anti-rabbit (LI-COR, Lincoln, NE, USA, 926-32211), IRDye 680RD goat anti-rabbit (LI-COR, Lincoln, NE, USA, 926-68071), IRDye 800CW goat anti-mouse (LI-COR, Lincoln, NE, USA, 926-32210), IRDye 680RD goat anti-mouse (LI-COR, Lincoln, NE, USA, 926-68070) for 1 h and subsequently imaged in a LI-COR Odyssey Infrared Imager (Lincoln, NE, USA).

### 2.8. Fractionation

Subcellular fractionation of cells was carried out as previously described [64]. Briefly, cell pellets were resuspended in cytosolic extract buffer (CEB; 250 mM sucrose, 70 mM KCl, 137 mM NaCl, 4.3 mM Na_2_HPO_4_, 1.4 mM KH_2_PO_4_) supplemented with 400 µg/mL digitonin (Sigma-Aldrich, St. Louis, MO, USA, D141-100MG), 100 µM PMSF (Carl Roth GmbH, Karlsruhe, Germany, S367.1), 10 µg/mL leupeptin (AppliChem, Darmstadt, Germany, A2183,0010) and 2 µg/mL aprotinin (Carl Roth GmbH, Karlsruhe, Germany, A162.1). The cytoplasmic fraction was removed after centrifugation, and the mitochondrial fraction was generated from the pellets via incubation in a mitochondrial lysis buffer (MLB; 50 mM Tris-HCl pH 7.4, 150 mM NaCl, 2 mM EDTA, 2 mM EGTA, 0.2% Triton X-100, 0.3% NP-40) supplemented with 100 µM PMSF, 10 µg/mL leupeptin and 2 µg/mL aprotinin. Extracts were centrifuged and the mitochondrial fraction was removed, before nucleic lysates were prepared from pellets in RIPA buffer, which contained HALT phosphatase inhibitors (Thermo Fisher Scientific, Waltham, MA, USA) and cOmplete proteinase inhibitors (Roche, Basel, CHE). The nuclear extracts were centrifuged to remove RIPA insoluble debris. Protein concentration in each fraction was quantified by BCA assay. Purity of the fractions was assessed via the presence of GAPDH in cytosolic fractions, HSP60 in mitochondrial fractions and LAMIN-A/C in nuclear extracts via quantitative immunoblots. 

Subcellular fractionation of cerebellar tissue was performed as previously described [65]. In brief, one cerebellum was first homogenised in Buffer A (150 mM NaCl, 50 mM HEPES pH 7.4; 1 M hexylene glycol) supplemented with 400 µg/mL digitonin, 100 µM PMSF, 10 µg/mL leupeptin and 2 µg/mL aprotinin using a pestle motor mixer. Samples were further homogenised via centrifugation through a QIAshredder (Qiagen, Venlo, The Netherlands). After a 10 min incubation period, samples were centrifuged to obtain the cytoplasmic fraction. Pellets were resuspended in Buffer B (150 mM NaCl, 50 mM HEPES pH 7.4, 1% NP-40, 1 M hexylene glycol) supplemented with 100 µM PMSF, 10 µg/mL leupeptin and 2 µg/mL aprotinin. Extracts were incubated for 30 min and centrifuged to generate mitochondrial fractions. Finally, pellets were incubated with 500 U benzonase nuclease (Sigma-Aldrich, St. Louis, MO, USA, E1014-25KU) to digest DNA. Nuclei were lysed via a 10 min incubation with Buffer C (150 mM NaCl, 50 mM HEPES pH 7.4, 1 M hexylene glycol, 0.5% sodium deoxycholate, 0.1% SDS) supplemented with 100 µM PMSF, 10 µg/mL leupeptin and 2 µg/mL aprotinin, and nuclear extracts were harvested as supernatant after centrifugation. The fractions were subjected to BCA assay for determination of protein concentration. Purity of fractions was again assessed via the presence of GAPDH in cytosolic fractions, HSP60 in mitochondrial fractions and LAMIN-A/C in nuclear extracts via immunoblotting.

### 2.9. Statistics

Data were statistically analysed using GraphPad Prism 8 Software. Grouped data were analysed via 2-way ANOVA followed by Sidak’s post-hoc test for multiple comparisons. Independent data were analysed via 1-way ANOVA followed by Tukey’s post-hoc test for multiple comparisons. Comparisons of two conditions were performed with unpaired *t*-test with Welch’s correction. Asterisks represent significance (* = *p* ≤ 0.05, ** = *p* ≤ 0.01, *** = *p* ≤ 0.001, **** = *p* ≤ 0.0001). *p*-values 0.05 < *p* < 0.10 were considered as a statistical trend (T) and are displayed as exact values. Data are displayed as mean ± standard error of the mean (SEM) with or without additional single values. Protein and transcript ratios are displayed as fold changes, relative to the untreated control condition.

## 3. Results

### 3.1. The Cerebellar Transcriptome Profile of ATM-Null Mice at 12 Months of Age

**Figure 1:**
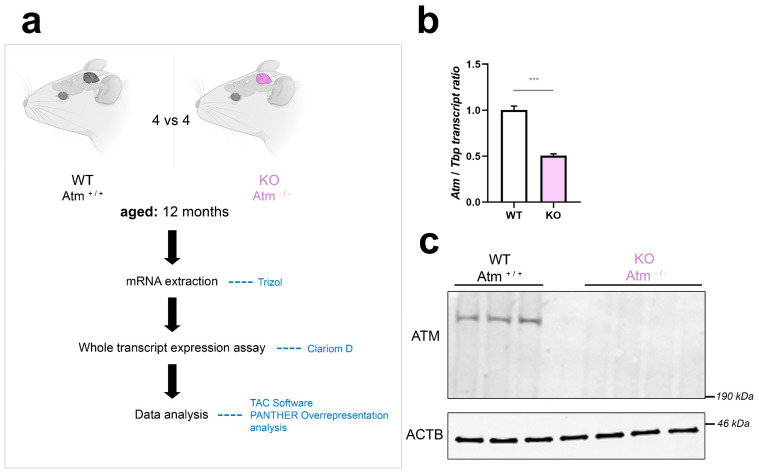
Workflow and quality control for genome-wide cerebellar transcriptome analysis from 12-month-old *Atm*-KO mice. (**a**) Schematic representation of the workflow performed in age and sex-matched *Atm*^+/+^ versus *Atm*^−/−^ mice (n = 4 vs. 4, two males and two females each, mutant selection based on reduced cerebellar weight). mRNA was extracted from cerebella of these animals using Trizol, and ClariomD microarray hybridisation was then performed. Data were analysed using the TAC Software provided by Affymetrix, and by PANTHER Overrepresentation analysis of pathway enrichments. (**b**) Mouse genotype validation via RT-qPCR, detecting the quantity of WT *Atm* transcript versus its reduction due to exon deletion and nonsense-mediated RNA decay in the *Atm*-KO samples, using *Tbp* transcript as normaliser (n = 4). (**c**) Mouse genotype validation via quantitative immunoblots, regarding ATM protein absence (3–8% Tris-acetate gels, lane 4 empty) versus beta-actin (ACTB) (4–20% Tris-glycine gel) as loading control (WT vs. *Atm*-KO, 3 vs. 4). Asterisks reflect significance: *** = *p* ≤ 0.001. Data are displayed as mean ± SEM.

As shown in Figure 1a, the global transcriptome analysis of cerebellar tissue was performed in four WT versus four ATM-null mice, aged in parallel until 12 months in pairs of identical sex. The genotype of analysed mice was controlled by breeding protocols, PCR from ear-punch DNA, RT-qPCR of *Atm* mRNA and quantitative immunoblots of ATM protein (Figure 1b,c). For the selection of appropriate animals, the cerebellar weight of KO samples was normalised versus WT of the same sex, and KO tissues with weight reductions until 50% were chosen. The global transcriptome profile of the ATM-null cerebellum is documented in Table S1. To ensure data reproducibility among different organisms in this strongly affected tissue at advanced age, we compared our ATM-null mouse cerebellar transcriptome profile at age 12 months with a published [66] proteome survey of A T patient cerebellar post-mortem samples (although distortions by altered tissue composition at end-stage will generate artefacts, and mass-spectrometry will detect maximally some 10,000 among all existing proteins), annotating the consistent findings in Table S1 and compiling these factors in Table 1. The comparison of our 12-month-old ATM-null mouse cerebellar transcriptome profile with previous A-T patient cerebrospinal fluid proteome data [67] revealed parallel reductions for *Reln*, *Fat2*, *Omd*, *Cntn6* (down) and *C4b* (up). This transcriptome was then interrogated in the context of known ATM functions and phenotypes, as far as they are known in the current literature. Given the scarcity of 12-month-old ATM-null mice with cerebellar anomalies, and in view of the massive widespread transcriptome changes observed (which are probably a direct consequence of altered phosphorylation cascades that alert to membrane stress and modulate nuclear transcription), we also performed extensive validation work in stressed cell models to elucidate the role of prominent molecular events.

For the factors with interspecies consistency and multi-omics reproducibility shown in Table 1, the transcript identity and average expression levels are shown in the first columns, followed by a heatmap of log2-fold-changes with up- (red colour) versus downregulations (blue), and false discovery rate *p*-values illustrated by a yellow colour. Prominent upregulations (grey) of neurofilament medium and light chain mRNAs (*Nefm* and *Nefl*) reflect the axon pathology at this disease stage and presumably represent cellular efforts to compensate the progressive neurofilament loss that is known to occur in A-T [6], while other upregulations probably represent efforts to substitute dendritic loss (*Map2*, *Tubb3*, *Tubb2a*) and cell adhesion (e.g., *Ncan*). Expression downregulations (green) concerned exclusively synaptic factors (*Dpysl4*, *Slc17a7*, *Cadps2*, *Syne1*, *Stxbp5l*) and might be due to impaired differentiation. 

**Figure 2:**
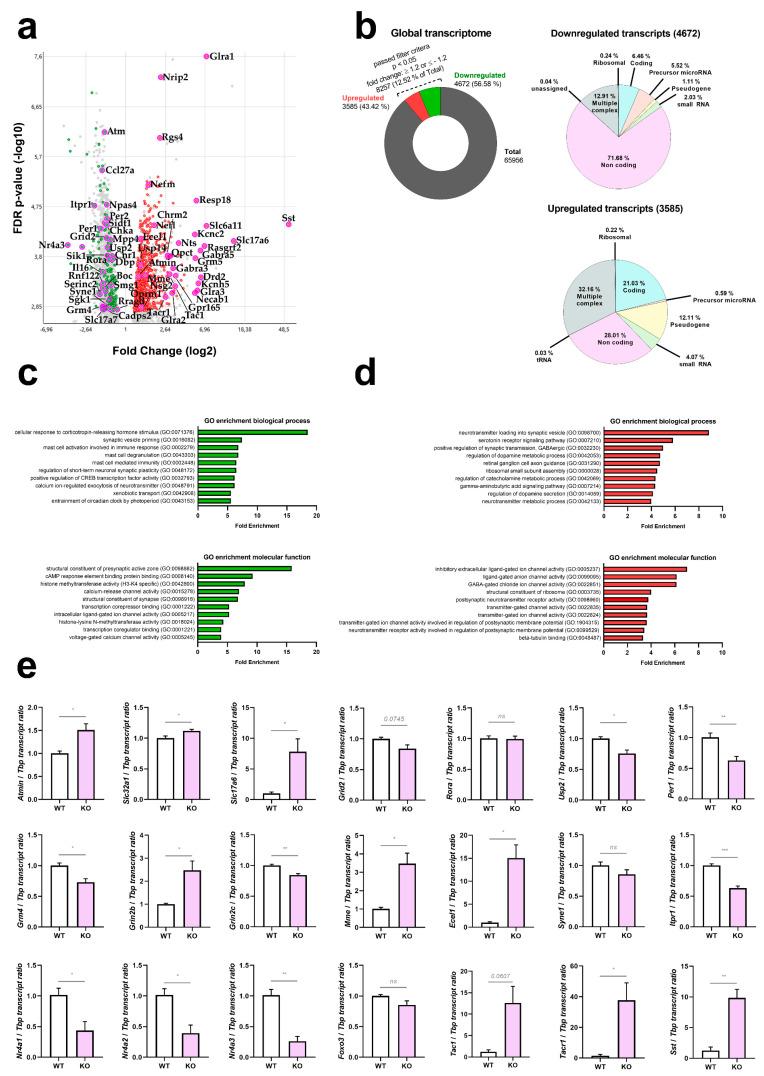
Genome-wide survey of transcript levels in cerebella from 12-month-old ATM-null mice. (**a**) Global transcriptome documentation via Clariom D microarrays, visualised as a volcano plot with symmetry due to logarithmic scales, where the *X*-axis shows down- versus up-regulations (in a green versus red colour, respectively) while the *Y*-axis shows the significance of changes via false detection rates (FDR), identifying factors with relevance for pathway enrichments and for follow-up studies by their gene symbols. (**b**) Total amount of detected transcripts (65,956) and ratio of transcripts that passed the filter criteria (8257, 12.52%). Of these, 43.42% (3585) were upregulated and 56.58% (4672) were downregulated. The upregulations and downregulation were further classified into different transcript categories, namely ribosomal, coding, precursor micro-RNA, pseudogene, small RNA, non-coding, tRNA and multiple complex, highlighting a prominent downregulation of non-coding RNAs. (**c**,**d**) Gene Ontology (GO) enrichment analysis of downregulated (green graphs) and upregulated (red graphs) transcripts, showing biological processes in the upper panel (prominent enrichment for cellular response to corticotropin-releasing hormone stimulus among downregulated transcripts, prominent enrichment for neurotransmitter loading into synaptic vesicle among upregulated transcripts), molecular functions in the lower panel (prominent enrichment for structural constituent of presynaptic active zone among downregulated transcripts, prominent enrichment for inhibitory extracellular ligand-gated ion channel activity among upregulated transcripts). (**e**) Dysregulation validation via RT-qPCR in these 12-month-old mouse cerebella (WT vs. *Atm*-KO, n = 4 vs. 4) for key factors in ATM interaction, excitability, neurotransmission and neuropeptide signalling. For statistical trends, the precise *p*-value was shown. Asterisks represent significance: * = *p* ≤ 0.05, ** = *p* ≤ 0.01, *** = *p* ≤ 0.001, ns = non-significant. Data are displayed as mean ± SEM.

In Figure 2a, a volcano plot displays the overall distribution of transcript dysregulations with actual significance (FDR < 0.05, corresponding to *p* < 0.0017), identifying particularly relevant coding transcripts by their gene symbols. As a noteworthy finding, ATM depletion was responsible for significant downregulations of mostly non-coding transcripts, whereas the upregulations concerned almost exclusively coding transcripts. The strongest downregulated microRNA was miR-495 (log2 fold change, FC −3.85, *p* = 0.0002) as angiogenesis-hypoxia-autophagy-synaptic depression modulator [68,69,70,71,72,73]. An even greater downregulation was detected for the non-coding RNA TC0500000412.mm.1 (FC −10.77, *p* = 6.30 × 10^−7^) as a prime example for the massive impact of ATM-loss on non-coding RNAs in general. The cellular roles of TC0500000412.mm.1 are unknown at present. In Figure 2b, pie charts reflect this massive contrast between non-coding downregulations versus coding upregulations, providing absolute numbers and percentages. Extreme upregulations of several factors that are selectively expressed in the choroid plexus, whose presence in the cerebellar samples was not controlled, were interpreted as artefacts. A bioinformatics survey of gene ontology terms in biological processes and molecular functions by PANTHER software indicated prominent deficits in corticotropin-dependent stress responses, as well as presynaptic machinery and vesicle priming (Figure 2c), versus prominent excess transcripts for neurotransmitter loading and channel activity (Figure 2d). The upregulations of neurotransmission components occurred without selectivity for any cell type, involving glutamate, GABA, glycin, muscarinic and nicotinic acetylcholine, as well as dopamine receptor transcripts. Notably, the neuropeptide signaling pathway (GO:0007218) was the 17th most enriched term among upregulations (FDR *p* = 2.02 × 10^−5^) and also showed a non-selective pattern in general, involving somatostatin, tachykinin, neurotensin, endothelin, vasohibin, encephalin, opioid mu and kappa3 signalling components. Furthermore, detailed bioinformatics studies showed significant enrichment on the STRING webplatform for ataxia genes, vesicular factors, calcium homeostasis factors, and immediate-early genes. The factors involved in these enrichments are annotated in Table S1, together with all ATM protein interactome components and the ATM kinase target proteins known at present.

In the disease context, the transcriptome showed a dysregulated expression with nominal significance for genes responsible for phenotypes of ataxia (compiled according to the Online Mendelian Inheritance of Man database, https://www.ncbi.nlm.nih.gov/omim/, accessed on 29 February 2022). Downregulations were observed for *Atm*, *Itpr1*, *Syne1*, *Grid2*, *Grik2*, *Fgf14*, *Rora*, *Gba2*, *Reln*, in good agreement with a previous proteome study of cerebrospinal fluid from A-T patients [67]*;* a significant enrichment was detected on the STRING webserver for “abnormal cerebellar granule neuron morphology” (q = 0.0014) for the cluster of ATM, RORA [74] and GRID2 [75] proteins; an enrichment for “postsynapse” (q = 0.0182) was detected for ITPR1 [66,67,75,76], SYNE1, GRID2 [75] and GRIK2 [77]; upregulations were observed for the ataxia genes *Mme*, *Ebf3*, *Vamp1*, *Ppp2r2b*, *Svbp*, without significant enrichment, but VAMP1 being a vesicle-associated factor like ATM. Significant expression changes existed also for genes responsible for the pathogenesis of telangiectasia (upregulation of *Sst*, *Sstr1*, *Sstr2*, *Tac1*, *Tacr1*, *Svbp*) [78,79,80,81], and for general growth (*Sst*, *Sstr1*, *Sstr2*) [82]. 

The significant dysregulation of ATM interactome components *Atmin*, *Nr4a1* and *Foxo3*/*Foxo1* (but not the ATM interactome components *Mre11*/*Rad50*/*Nbs1*, nor its downstream effectors *Chk2* and *Tp53*) argued against neural ATM functions at this cerebellar age in DNA damage repair, instead suggesting osmotic/oxidative/nutrient stress [83,84,85]. Interestingly however, the deubiquitinase USP2 was reported recently to function in the ATM/NBS1 interactome [86], and showed strong downregulation within the ATM-null cerebellar transcriptome. Even the relatively weak *Kat5* induction observed may be relevant, in view of the known role of KAT5-dependent ATM Lys3016 acetylation. 

Finally, among previously reported ATM phosphorylation target proteins [87,88,89] with significant dysregulation (see Table S1 annotations) in the 12-month-old ATM-null mouse cerebellar transcriptome, RTN4 (NOGO-A), DOCK10, FSCN1, SOX10, SEPT9 and CCNL2 were already implicated in glutamatergic synapse and dendrite effects [90,91,92,93,94,95]. Unexpectedly, the transcript upregulations concerned all neurotransmitter and neuropeptide pathways, rather than a signalling balance between glutamate-excitation on the one hand versus GABA-inhibition on the other hand [39]. 

Validation experiments by the independent method RT-qPCR in the remaining cerebellar tissue from these 12-month-old ATM-null and WT mice confirmed these dysregulations for practically all factors studied. These experiments focused on ATM interactors, ataxia genes, neurotransmitter loading factors, glutamate receptors, immediate early response components, and neuropeptide signalling molecules (Figure 2e). The RT-qPCR validation of these selected dysregulations was extended to cerebellar tissue from 1.5–3-month-old ATM-null versus age-/sex-matched WT mice, showing similar dysregulations to occur early on for *Nr4a1*, *Nr4a2*, *Oprm1*, and *Tacr1* (Figure S1). Furthermore, the 12-month-old ATM-null cerebellar transcriptome confirmed previous RT-qPCR results in an ATM-null cerebellum at the age of 2 months [67] regarding the downregulations of *Itpr1*, *Atp2b2* and *Grin2c*, versus the upregulations of *Grin2b* and *Cyp46a1* mRNA levels.

### 3.2. In Human Neural Cells with Stable ATM-Knockdown, Cerebellar Hallmark Dysregulations Are Recapitulated Best after Osmotic Stress, and Partially after Trophic Stress

ATM-deficiency was studied further in cell culture, to assess the reproducibility of these findings in humans, and to identify the most suitable stressor in vitro that mirrors such age-associated dysregulations, while enabling us to generate unlimited samples for mechanistic studies. We used the human SH-SY5Y neuroblastoma cell line, introduced various ATM shRNAs via lentiviral transduction, and produced a stable *ATM*-KD cell line that achieved high ATM protein- and mRNA-reduction for further analysis. The most efficient KD was produced by shRNA#2 (hereafter be referred to as shATM), triggering obvious changes in cellular morphology (Figure S2a), and reductions of *ATM* transcript to 36% (Figure S2b) and protein to 9.5% (Figure S2c), compared to the non-target shRNA control (NT CTRL) condition. In this human neural cell line, the application of the osmotic stressor chloroquine (CQ) did not alter the abundance of ATM protein, but induced phosphorylation at ATM residue S1981 (1.8-fold, with *p* = 0.1408 in three biological replicates), an expected event for DDR-triggered autophosphorylation/activation of this stress sensor molecule (Figure S2d). 

**Figure 3:**
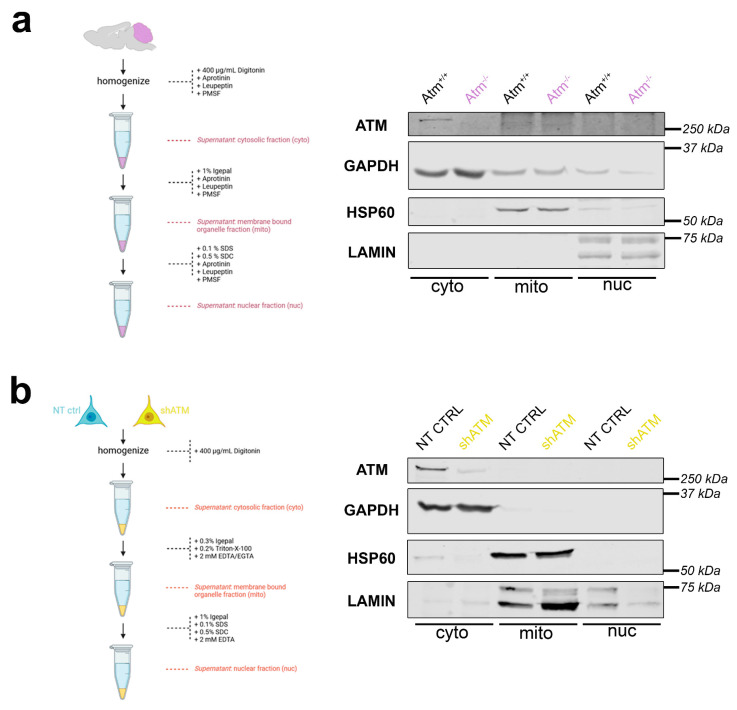
ATM localisation in subcellular fractions by differential detergents. The workflow scheme is shown on the left side. On the right side, samples represent cell fractions of cytoplasm (cyto), mitochondria (mito) and nucleus (nuc). The purity of each aliquot was assessed by the markers GAPDH for cytoplasm, HSP60 for mitochondria, and LAMIN-A/C for nucleus. The size markers in kilodaltons (kDa) on the right margin of each gel confirm the expected molecular weight of each protein studied. (**a**) Immunoblot detecting ATM in WT versus *Atm*-KO mouse cerebella from 3.5-month-old mice. (**b**) Immunoblot detecting ATM in non-target shRNA transduced control (NT CTRL) versus shATM transduced mutant SH-SY5Y neuroblastoma cells.

To assess whether SH-SY5Y neuroblastoma cells have a similar distribution of ATM in subcellular fractions as adult cerebellar tissue, differential detergent isolation of nuclear, mitochondrial, and cytoplasmic fractions was performed firstly in cerebella from WT versus ATM-null mice at the age of 3.5 months (Figure 3a), and secondly in SH-SY5Y NT CTRL cells compared to shATM cells (Figure 3b). Even though there was some leakage from the GAPDH-immunopositive cytoplasmic (cyto) fraction to the HSP60-positive mitochondrial (mito) and the LAMIN-A/C-positive nuclear (nuc) fraction, ATM was clearly located in the cytoplasmic rather than the nuclear fraction in the mouse cerebellum (Figure 3a). This finding is novel, since previous analyses until maximal cerebellar age of 6 weeks, after completion of Purkinje neuron maturation and granule cell precursor migration [96,97,98], had observed ATM more in nuclear than in cytosolic fractions. 

A localisation in the cytosolic fraction was also clearly observed for ATM in SH-SY5Y cells, although the gels exhibited some leakage of the nuclear fraction to the mitochondrial fraction (Figure 3b). Importantly, this cytoplasmic localisation of ATM in vitro was not altered by administration of the osmotic stressor CQ or the genotoxic stressor bleomycin (BLEO) in several independent experiments (Figure S3a,b). One experiment with LY stress, and one experiment with NaARS stress, also failed to detect an ATM localisation change. These results indicate that the *ATM* knockdown in SH-SY5Y neuroblastoma cells can be used as useful in vitro models for neural *Atm*-deficiency, regarding transcript and protein levels, stress induction and subcellular fractionation.

### 3.3. The ATM-Null Cerebellar mRNA Dysregulations Are Mimicked in SH-SH5Y Cells by ATM Knockdown Rather Than ATM Kinase Antagonism, and by CQ Better Than Trophic/Oxidative/Genotoxic Stress

**Figure 4:**
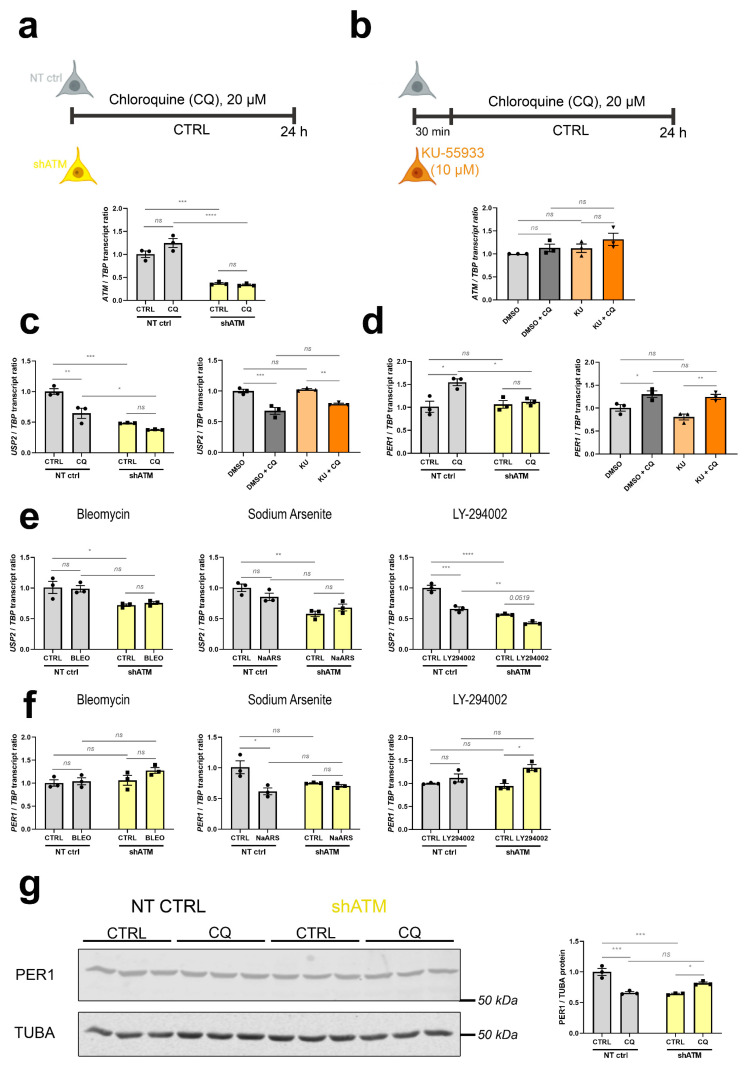
*ATM*-KD successfully models the dysregulations found in vivo, while inhibition of ATM kinase function is not effective. (**a**) Scheme of chloroquine (CQ) stressor treatment in *ATM*-KD SH-SY5Y cells (upper panel) and corresponding *ATM* transcript levels determined via RT-qPCR (n = 3, lower panel). (**b**) Scheme of CQ stressor treatment in parental SH-SY5Y cells with KU-55933 ATM kinase inhibitor pretreatment (upper panel) and corresponding *ATM* transcript levels determined via RT-qPCR (n = 3, lower panel). (**c**) Comparison of ATM interactor *USP2* mRNA and (**d**) immediate early transcript *PER1* levels in *ATM*-KD (left) vs. ATM-kinase-inhibited parental cells (right) after CQ stress, as determined via RT-qPCR (n = 3). (**e**) Reduction of *USP2* transcripts upon CQ-stress and in *ATM*-KD was largely reproduced by LY-294002 stressor treatment, but not after BLEO and NaARS treatment of *ATM*-KD cells (n = 3). (**f**) BLEO, NaARS and LY-294002 stressor treatments did not reproduce the ATM dependent *PER1* transcript induction present in aged ATM-null cerebellum, which was however successfully modelled after CQ-stressor treatment of *ATM*-KD cells (n = 3) as seen in panel (**d**). (**g**) PER1 protein was reduced by CQ-stress administration in NT CTRL cells and generally in shATM cells, as determined by quantitative immunoblots (n = 3). For statistical trends, the precise *p*-value was shown. Asterisks display significance. * = *p* ≤ 0.05, ** = *p* ≤ 0.01, *** = *p* ≤ 0.001, **** = *p* ≤ 0.0001, ns = non-significant. Data are displayed as bar plots with data points, mean ± SEM.

To understand whether the cerebellar dysregulations of old ATM-null mice are due to ATM absence as a platform for protein complex formation, or absent ATM kinase activity, we assessed if they are recapitulated after stress in neuroblastoma cells upon KD of *ATM* mRNA, or after treatment with KU-55933 (KU) as a pharmacological inhibitor of ATM-mediated phosphorylation (scheme and control of *ATM* mRNA levels in Figure 4a,b). As representative transcripts under control of ATM, we chose upstream effectors such as *USP2* in view of its role within the ATM-interactome, and *PER1* as immediate-early transcript modulated by phosphorylation cascades (Figure 4c–f). Figure 4c shows the expected significant downregulation of *USP2* after CQ administration in the *ATM*-KD cells (to 65% of control after CQ, and further reduction to 48% and 38% in shATM cells with and without CQ-stress), while in KU-treated cells downregulation of *USP2* was only generated by CQ-treatment but not the kinase inhibition. Similarly, Figure 4d shows a significant ATM-dependent CQ stressor effect, with the significant 1.5-fold induction of *PER1* by CQ stressor being abolished to control levels in shATM cells. Again, this effect was not reproduced in KU-treated cells. For *USP2* (Figure 4e), genotoxic and oxidative stress were unable to trigger the downregulation; only trophic stress via treatment with the PI3K-inhibitor LY-294002 resulted in a significant ATM-dependent reduction. For *PER1* (Figure 4f), all other stressors were ineffective. Exploiting the availability of a specific and sensitive anti-PER1 antibody, a reduction of PER1 protein was found in neuroblastoma cells with stable *ATM*-KD even before the application of acute stress (Figure 4g). The administration of CQ resulted in a PER1 reduction in NT CTRL cells, but a converse PER1 protein induction in shATM cells. Thus, a combination of *ATM*-KD with CQ-stress appeared to represent the best in vitro modelling approach in SH-SY5Y neuroblastoma cells, to investigate the roles of cerebellar mRNA dysregulations in aged ATM-null cerebellum.

### 3.4. Also in Human Cells, ATM-Deficiency Impacts Key Pathomechanism Factors Like Interactor ATMIN, Immediate-Early mRNA FOXO3, Osmotic Regulator RRAGD, Vasoconstriction Regulator ECEL1, and Ataxia Transcripts GRID2, ITPR1 and MME

Although the global transcriptome profile of an old ATM-null cerebellum identified many novel pathogenesis events, it remained unclear to what degree these findings are conserved in humans, and whether they can be explained by osmotic stress. Therefore, validation experiments were conducted with RT-qPCR and quantitative immunoblots to assess key factors in human SH-SY5Y cells with *ATM*-KD, unstressed or after CQ administration. For validation of individual dysregulations, we selected crucial effectors of ATM function and critical determinants of the phenotypes that characterise A-T. 

**Figure 5:**
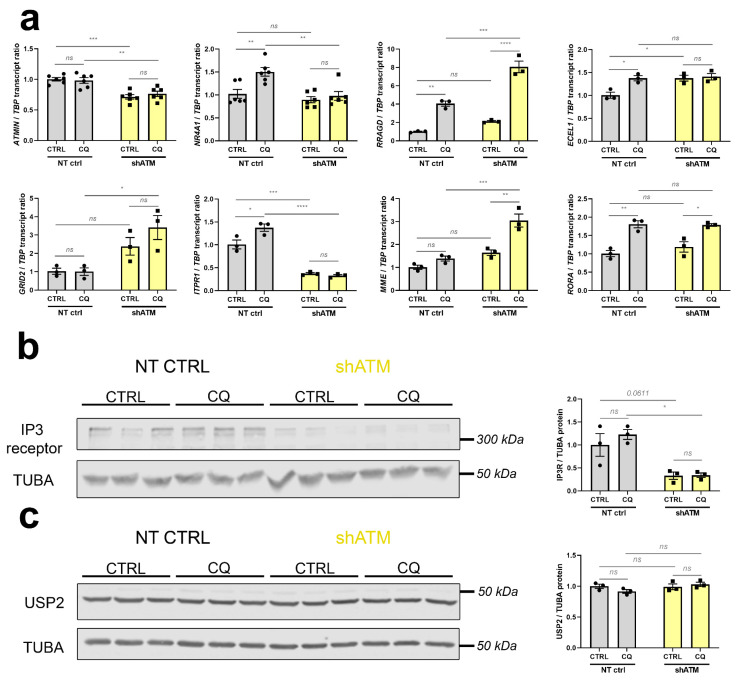
The stressor chloroquine provides the most effective model in SH-SY5Y *ATM*-KD cells, for representative strong dysregulations in different pathways, which were previously documented in aged ATM-null cerebella. (**a**) *ATMIN* mRNA (n = 6) was analysed as interactome component of ATM. *NR4A1* (n = 6) was analysed as immediate early gene. *RRADG* (n = 3) transcript levels were analysed as positive controls for osmotic stress elicited by CQ. *ECEL1* (n = 3) and *MME* (n = 3) were analysed for the group of neuropeptide endopeptidases. mRNAs for *GRID2* (n = 3), *ITPR1* (n = 3) and *RORA* (n = 3) were analysed as known ataxia disease genes. (**b**) The protein Inositol-1,4,5-Trisphosphate Receptor (IP3R, encoded by *ITPR1*) was also significantly reduced in shATM cells compared to NT CTRL cells as determined by quantitative immunoblots, while induction by CQ stressor treatment did not reach significance (n = 3). The double band around 315 kDa was quantified by densitometry. Tubulin A (TUBA) was used as sample loading control and normaliser, in view of its high abundance similar to IP3R. (**c**) USP2 protein appeared unchanged in quantitative immunoblots (n = 3) of shATM cells compared to NT CTRL cells, despite the transcript induction shown in Figure 3e. For statistical trends, the precise *p*-value was shown. Asterisks reflect significance: * = *p* ≤ 0.05, ** = *p* ≤ 0.01, *** = *p* ≤ 0.001, **** = *p* ≤ 0.0001, ns = non-significant. Data are displayed as bar plots with data points, mean ± SEM.

In parallel to the desired reduction of *ATM* in SH-SY5Y knockdown cells documented in Figure 4a, these further studies (see Figure 5 and Figure S4) confirmed strong genotype-dependent downregulations for *ATMIN* mRNA (to 71% and 76%) as a mediator of ATM responses to osmotic and oxidative stress. *NR4A1* and *FOXO3* mRNA, as immediate-early mediators of phosphorylation signals to the nucleus, were both found to be responsive to CQ-stressor treatment (1.5-fold and 1.6-fold increase, respectively), while displaying abrogated induction in shATM cells (Figure 5a, Figure S4a). In addition, the CQ-triggered inductions of calcium-dependent kinases *CAMK2A* and *CAMK4* mRNA (4.1-fold and 1.3-fold, respectively) were significantly impaired upon *ATM*-KD (Figure S4a). Importantly, an ATM-dependent mRNA downregulation (to 32% and 37% for unstressed and stressed conditions, respectively) was also observed for the ataxia gene *ITPR1* and might therefore be interpreted as a loss-of-function that may have a primary role in the pathogenesis of autosomal recessive A-T, while the other ataxia genes *GRID2* [99,100] and *MME* [101] showed ATM-dependent mRNA upregulations (2.4-fold and 3.4-fold for *GRID2*; 1.6-fold and 3.0-fold for *MME*, in unstressed and stressed *ATM*-KD cells) that may represent compensatory efforts, and the ataxia transcript *RORA* [102,103] exhibited only a response to osmotic stress (1.8-fold increase in CQ treated cells; Figure 5a). As further evidence for compensatory reactions to osmotic stress, *RRAGD* mRNA (encoding Ras-related RagD amino acid sensor [104,105]) showed significant upregulation after CQ treatment, and even bigger upregulation after *ATM*-KD (4.0-fold induction in NT CTRL cells and 8.1-fold increase in stressed shATM cells, Figure 5a). In addition, *SGK1* transcript induced only upon CQ treatment in shATM cells (1.4-fold, *p* = 0.3903 and 2.4-fold, *p* = 0.0007) corroborates the presence of osmotic stress (Figure S4a). As a putative modifier of vasodilatation, *ECEL1* mRNA [106,107] was found upregulated after CQ stress in NT CTRL cells (1.4-fold, Figure 5a) and after oxidative stress in *ATM*-KD cells (1.4-fold), as well as after CQ stress during ATM kinase inhibition (1.2-fold) (Figure S4b).

Quantitative immunoblots were conducted when commercial antibodies were available to us with sufficient specificity and sensitivity to detect the endogenous protein levels. For the human IP3R protein encoded by *ITPR1* transcript, these experiments confirmed a strong reduction of abundance (to about 35%) upon ATM-deficiency (Figure 5b). In contrast, the quantitative immunoblots indicated the protein levels of USP2 to be unchanged by CQ and by *ATM*-KD (Figure 5c), so apparently the significant *USP2* mRNA reduction after CQ and *ATM*-KD demonstrated in Figure 4c does not rapidly impact steady-state immunoreactivity, and it may be that posttranslational control of USP2 and its MDM4/HDMX-MRN-complex-association [86,108,109] are more decisive for short-term USP2 activity regulation than its resynthesis. Still, our mRNA findings confirm USP2 as a very consistently ATM-dependent factor whose expression is modified by cytosolic ATM in neural cells, and which functions very upstream in the ATM-dependent stress response pathways—so it might be a useful target of novel preventive therapies.

### 3.5. ATM-Null Mouse Cerebellum Alternatively Spliced mRNAs Are Enriched for Neurotransmission and Neuropeptide Signalling Factors

**Figure 6:**
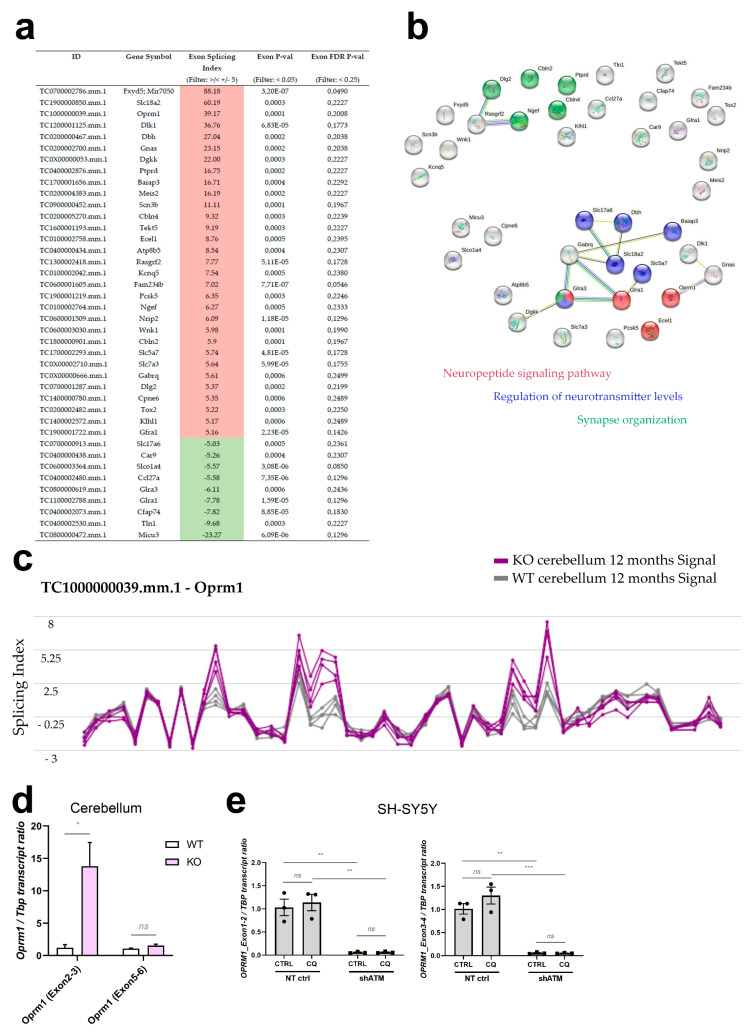
Genome-wide survey of alternative splicing in cerebella from 12-month-old ATM-null mice reveals enrichment for neuropeptide signalling pathways and neurotransmission. (**a**) Table of all transcripts that display excessive alternative splicing (Filter criteria: Exon Splicing Index >/< +/−5, Exon *p*-value < 0.05, Exon FDR *p*-value < 0.25 and Group: Multiple Complex and Coding). Increased exon-splicing index is highlighted in red, decreased exon-splicing index in green. (**b**) STRING functional connection networks (https://string-db.org/, accessed on 6 April 2023) of these alternatively spliced transcripts. Red buttons belong to the neuropeptide signaling pathway, blue buttons are implicated in regulation of neurotransmitter levels, and green buttons exert functions during synapse organization. (**c**) Structure view of the *Oprm1* transcript structure with splicing indexes displayed in line plots for WT (grey) and *Atm*-KO (purple) cerebellum. Validation experiments in (**d**) cerebellum of 12-month-old *Atm*-KO vs. WT (4 vs. 4) mice and (**e**) SH-SY5Y *ATM*-KD cells under CQ-stress. Asterisks reflect significance: * = *p* ≤ 0.05, ** = *p* ≤ 0.01, *** = *p* ≤ 0.001, ns = non-significant.

For rapidity, stress responses are conveyed through the cytoplasm by phosphorylation signals to adapt transcription of immediate-early genes in the nuclear chromatin, but even more directly stress signalling readjusts the alternative splicing of existing transcripts in the nucleus and the editing of actively translated transcripts in the cytosol. Since the ClariomD microarrays employed for cerebellar transcriptome profiling represent almost all exons, we also assessed splice dysregulations in this dataset from aged ATM-null mice, employing the “Alt Splice View” function in the Transcriptome Analysis Console from AppliedBiosystems. Interestingly, upon filtering for significant changes (*p*-value < 0.05, FDR *p*-value < 0.25) with strong fold-change (Exon Splicing Index >/< +/−5), a set of 40 alternatively spliced transcripts was identified. Of these, 31 displayed an overall increased exon-splicing index, while 9 showed a reduced exon-splicing index (Figure 6a). More detailed assessment revealed that most of them represent altered quantities of a single exon within a dysregulated transcript, and because of concerns that not all oligonucleotide probes within an mRNA can be expected to exhibit parallel linear signal changes and might therefore mimic alternative splicing artificially, we decided to annotate such observations for the main candidates (Table S1, second datasheet). Again, the neuropeptide signaling pathway (FDR q = 1.16 × 10^−6^), regulation of neurotransmitter levels and synapse organization were prominent, as identified on the STRING Web browser (Figure 6b). 

*Oprm1* stood out with an overall exon-splicing index of 39.17, which is summarised graphically (Figure 6c) by the Transcriptome Analysis Console (Applied Biosystems) for each oligonucleotide probe along the transcript structure. Extrapolating probes within individual exons or at the junction between known exons or cryptic exons from these data in mouse onto known facts in human, we tested the credibility of these *Oprm1* splice changes by RT-qPCR in human neuroblastoma cells. It should also be taken into account that an oligonucleotide probe within an exon may show a differential increase or decrease, while a Taqman assay is usually quantifying the amplification product at the junction between two adjacent exons, so a splice change may be detectable by a specific RT-qPCR assay but not by the neighbouring assay, and the effects may not be conserved between species, with differing exon number nomenclature. As shown in Figure 6d, the exon 2–3 boundary of *Oprm1* exhibited a 15-fold signal increase in old ATM-null cerebellum, while the exon 5–6 junction signal was unaltered. In the human SH-SY5Y cells, the RT-qPCR results of exon 1–2 and exon 3–4 boundaries in *OPRM1* transcript also showed a massive dysregulation upon ATM deficiency (reduction to 5%), with no significant change after osmotic stress (Figure 6e). This downregulation was not detected upon ATM kinase inhibitor treatment (Figure S4c), where *OPRM1* transcript levels remained stable. *OPRM1* transcript downregulation exclusively in the *ATM*-KD condition was robustly reproduced also in experiments with BLEO, NaARS and LY-294002 (Figure S4c), reflecting stressor-independent effects of ATM deficiency itself. Thus, although the splicing details may differ between species and cell type, with cerebellar tissue even revealing opposite effects than cultured neuroblastoma cells, the mouse microarray data and validation experiments by RT-qPCR in mouse cerebellum and human neuroblastoma cells clearly identified the opioid mu receptor as mRNA under control of ATM.

## 4. Discussion 

Overall, the novel transcriptome profile of 12-month-old ATM-null cerebellum has shed light on the primary role of osmotic stress in A-T pathogenesis, identified molecular correlates of A-T phenotypes such as incipient ataxia/vasodilatation/growth impairment, defined dysregulations of interactor molecules of cytosolic ATM that may represent useful upstream targets of neuroprotection, and documented generalised affection of neurotransmission and neuropeptide signalling—presumably mediated by cytoplasmic vesicles that have ATM protein associated to them. Given that the validation experiments were able to reproduce faithfully many dysregulations in human cell culture models of A-T, it is worthwhile to take all cerebellar dysregulations seriously and discuss the novel evidence extensively.

### 4.1. The Cerebellar Transcriptome Profile of ATM-Null Mice at 12 Months of Age

This cerebellar transcriptome profile was exceptionally informative, probably because protein kinases such as ATM are part of phosphorylation cascades that relay information on membrane events to the nucleus, governing transcriptional responses to stimuli versus stress. Before experiments in a cell culture model of ATM dysfunction validated whether individual dysregulations are reproducible in human, and how they depend on stress, it is important to understand the relevance of these key factors within the complex pathogenesis of cerebellar A-T. Thus, we feel the necessity to discuss the integration of all the strong dysregulations with multiple weaker effects within the same significantly enriched pathway, because often upstream events are small, while subsequent signalling cascades will amplify the fold-changes of downstream molecular events. After identifying relevant changes in upstream coordinators and mechanisms of each affected pathway, it is important to explore how they are connected to ATM and how they overlap with other cerebellar ataxias. This discussion text aims to describe a coherent scenario where the failure of stress responses and the underlying toxic agents can be better understood. 

To comprehend these observations, it may help to consider the analogies between the cerebellar pathology in A-T on the one hand, versus the common sunburn on the other hand. Ionising radiation and ultraviolet-B (UVB) light are typical causes of DNA-DSB in the nucleus, which are sensed by ATM to coordinate repairs. UVB is also the typical cause of sunburns in skin tissue, where not only DNA-DSB is known to ensue, but also cytoplasmic effects like calcium-dependent excitation with chemokine/cytokine release, vasodilatation, inflammation, pain, and keratinocyte death or carcinogenesis [110,111]. It is already known that ATM is needed after sunburns to mitigate UVB damage and restore normal cell growth [112,113,114], so ATM deficiency is indeed expected to impact on cytoplasmic homeostasis, including prolonged vasodilatation, oedema and pain via peptide-signalling. The present transcriptome data provide the molecular details for a similar scenario of pathomechanism in cerebellar tissue. 

Regarding the prominent neurotransmission effects of cytosolic ATM, previous investigations had reported it to be key for glutamatergic excitation, while ATR was implicated in a complementary role for inhibitory GABAergic neurotransmission [39]. Indeed, our hypothesis-free microarray profiling observed a widespread profound affection of the glutamatergic pathway, reflected by downregulations of receptors *Grid2* (which is responsible for Spinocerebellar Ataxia type 18 [115]), *Grid2ip* [116], *Gria4*, *Grik2*, *Grin2c*, *Grm1* (responsible for autosomal recessive Spinocerebellar Ataxia type 13, and autosomal dominant Spinocerebellar Ataxia type 44 [117,118]) and *Grm4*, the glial high affinity glutamate transporter *Slc1a3* (encoding EAAT1/GLAST which is responsible for Episodic Ataxia type 6 [119,120]) and *Slc1a6* (encoding EAAT4 which is involved in Spinocerebellar Ataxia type 5 [121,122], the mitochondrial glutamate transporter *Slc25a22*, and an eye-catching contrast between downregulation of transporter *Slc17a7* (encoding VGLUT1 in parallel fibres of the cerebellar cortex), versus massive upregulation of *Slc17a6* (encoding VGLUT2 in climbing fibres of the deep cerebellum) [123]. Glutamatergic upregulations also affected *Grm5*, *Grm3*, *Grm8*, *Grin2b*, *Grin3a*, *Grid1*, *Slc1a1* (encoding EAAT3), *Slc1a2* (encoding EAAT2/GLT1), *Slc1a4* (encoding ASCT1), the AMPA-receptor interactor *Nsg2* [124], the glutamate receptor interactor *Grip1* [125], and excitation-repressing *Cnr1* [126]. 

While extending our notions about a glutamate-focused ATM role, this transcriptomic approach permitted the additional insight that ATM loss also upregulates receptors in the inhibitory GABA-pathways (*Gabra3*, *Gabra5*, *Gabra2*, *Gabrg1*, *Gabrg3*, *Gabrg2*, *Gabrq*, *Gabre*, *Gabrb1*, *Gabarapl1* (contrasted by downregulation only for *Gabra6*), together with upregulation of GABA-transporters *Slc6a11* and *Slc32a1*, as well as receptors in the inhibitory glycine (*Glra1*, *Glra3*, *Glra2*, *Glra4*), and in the dopamine (*Drd2*, *Drd5*), acetylcholine (*Chrm2*, *Chrna4*, *Chrm3*, *Chrna7*, *Chrna6*, *Chrm5*, *Chrnb4*, *Chrnb3*) systems. These data suggest that ATM plays a main role in the stress adaptation of synaptic vesicles not only for excitatory but also for inhibitory signalling, possibly via the regulation of vesicle availability/loading/release/recycling. 

Regarding neuropeptides and their receptors, additional general affection of these signalling pathways was documented, with upregulations of somatostatin (*Sst*, *Sstr1*, *Sstr2*), neurotensin (*Nts*, *Ntsr1*, *Ntsr2*), tachykinin (*Tac1*, *Tacr1*, *Tacr3*), neuropeptide-Y (*Npy*, *Npy1r*, *Npy4r*) mRNAs, and *Resp18* mRNA encoding a factor responsible for neuropeptide packing in dense core vesicles [127,128,129], as well as *Qpct* encoding a factor responsible for the N-terminal pyroglutamyl residues of neuropeptides and cytokines [130]. In addition, *Oprm1* as mu-type (morphin-type), *Oprl1* as kappa3-type (nociception-type), and *Oprk1* as kappa1-type (for alpha-neoendorphins and dynorphins) of opioid receptors showed upregulated transcripts. These cerebellar findings identify molecular mechanisms that show how ATM deficits trigger not only excessive changes in neurotransmission, but may come to impact vasodilatation/telangiectasia and inflammatory oedema (via tachykinins, *Ecel1*), growth and fertility (via somatostatin and neuropeptide-Y), immunity and lipid metabolism (via neurotensin), as well as pain perception (via opioids). As one of the strongest upregulated transcripts, *Rgs4* is a known regulator of G-protein signalling downstream from mu- and kappa-opioid signalling [131,132,133,134]. Even the neuropeptide activator *Pcsk1* and its inhibitor *Pcsk1n*, as well as neuropeptide inactivators like *Mme* (which is responsible for Spinocerebellar Ataxia type 43 [101]) and *Ecel1* were upregulated. These data suggest that ATM plays a stress adaptor role in general also for dense core vesicles where neuropeptides are stored. 

Regarding the atrophy of the aged cerebellum in A-T, it is plausible to pay attention to neurotrophins and other cytokines, which are stored in large dense core vesicles (LCDV) before their release and where ATM might play a similar role as for neuropeptides. Indeed, upregulations of neurotrophin receptors *Gfra1* and *Gfra2* [135,136], inhibitory neurite growth modulators *Slitrk3* [137,138], *Slitrk5* [139] and *Slitrk6* [140], neuronal sorting receptors *Sorcs1* and *Sorcs2* [141,142,143,144], stress-dependent transcription factor *Jun* with its kinase *Mapk9*/*Jnk2* [145,146], and heavy-metal-toxicity-inducible death executor *Ngfrap1* [147,148] suggest at first glance that LCDV pathology might contribute to a trophic imbalance in the cerebellum. Furthermore, a downregulation of the ligands *Nrg1* and *Nrg3* with converse upregulation of their receptor *Erbb3* and *Erbb4* transcripts was observed [149]. Crucial downregulations of neurotrophin *Ntf3* with its receptor *Ntrk3*, as well as the sorting receptor *Sorl1* [150], have to be noted. However, while the systematic interrogation of neurotrophins confirmed mostly upregulations as in neurotransmission and neuropeptide pathways, a similar systematic interrogation of cytokine receptors and their ligands did not reveal a similarly uniform effect. On the one hand, increased transcript levels were documented for *Tgfbr1*, *Tgfbr2*, *Pdgfra* (with ligand *Pdgfc*), *Epha4*, *Epha5*, *Epha5* (with ligands *Efna2*, *Efna3*, *Efna5*), *Fgfr3* (with ligands *Fgf18* and *Fgf5*), *Fgfr1op2*, *Csf1r*, *Bmpr1b*, *Lifr*, *Atp2b4*, *Tnfrsf13c*, *Tnfrsf21*, *Fzd8*, *Sfrp5*, *Kdr*, and ligands *Bmp2*, *Fgf13*, *Efnb3*, *Il18*, *Il33*, *Il34*, *Igf1* without their receptors. On the other hand, a smaller number of conversely decreased transcript levels were documented for *Fzd4* (with downregulated ligands *Wnt3*, *Wnt7a*), *Igf2r* (with downregulated ligand *Igf2*), *Igflr1*, *Epha3*, *Pdgfrl*, *Sfrp1*, *Kit*, *Socs7*, *Il20rb*, and ligands *Bmp1*, *Bmp7*, *Fgf14*, *Il16* without their corresponding receptor. Thus, while a systematic effect of ATM on all cytokines and neurotrophins is doubtful, specifically the deficits of *Ntf3* and *Ntrk3* are relevant for the survival of cerebellar granule neurons in a mechanism via Phospho-inositol-3′-kinase (PI3K). Importantly, the vesicle release of neurotransmitters, neuropeptides and neurotrophin-3 has a common upstream mediator in *Cadps2*, which showed decreased cerebellar mRNA levels [151,152,153,154,155,156]. The balance between neurotrophin support and glutamate neurotoxicity is known to be critical also for the survival of Purkinje neurons [157,158,159,160]. 

What other pathways were impacted in several components across the cerebellar transcriptome profile of ATM-null mice, given that the hypothesis-free global transcriptomics approach might give novel clues to understanding A-T pathogenesis better? Second messengers downstream from neuropeptide receptors appeared altered, in view of the G-protein signalling factors *Rgs4*, *Rasgrf2* and *Gpr165* upregulations [131,161,162], and the dysregulation of calcium modulators *Necab1* (up) [163] versus *Itpr1* (down, its loss-of-function being the cause of autosomal dominant Spinocerebellar Ataxia types 15 and 29, as well as autoimmune cerebellar ataxia) [57,164,165,166]. The decreased mRNA levels of inositol-trisphosphate receptor *Itpr1*, and of *Cadps2* (the Ca^2+^-dependent release activator for neurotransmitters, neuropeptides and neurotrophins), might be underlying contributors to this generalised pathology, given that loss-of-function of both downregulated factors results in cerebellar ataxia [154,164,167,168,169]. As further coordinators of pathology that are potentially upstream, the deficits of inositol-triphosphate-associated *Astn2*, *Sorl1*, and *Mpp4* levels could lead to inappropriate localisation of membrane proteins away from the tip of neural processes [170,171]. 

Regarding upstream factors within the protein interactome of ATM, the following dysregulations deserve discussion: As a key modulator much farther upstream in the pathomechanism, the increased levels of ATM interactor *Atmin* mRNA probably represent a compensatory response to ATM dysfunction, possibly affecting synaptic adhesion [172,173]. The *Atmin* upregulation upon ATM deficiency was unexpected, since both factors were thought to stabilise each other, with ATMIN levels being reduced upon ATM decrease, and vice versa [60,83]. The ATM-interacting MRN complex responsible for DNA damage signalling did not show any changed mRNA levels, but the NBS1 stabilising factor *Usp2* mRNA displayed a downregulation of similar effect size and significance as *Atm* mRNA [86]. USP2-null mice show impaired motor coordination and balance [174], so its deficiency in an ATM-null cerebellum might contribute to ataxia pathogenesis. This deubiquitinase is also known as a regulator of circadian clock components [175], and indeed several USP2 effectors also showed deficient transcript levels, such as *Cry1* and *Cry2.* In view of the role of KAT5 for the regulation of ATM activity, it may also be relevant that a transcript reduction in our dataset was observed for the KAT5-dependent kinase *Chka*, which is responsible for phospholipid biosynthesis [176,177]. 

Overall, excitation and growth stimuli in the ATM-null mouse cerebellum appeared to elicit deficient nuclear responses, in view of the downregulation of immediate-early genes *Nr4a3*/*Nr4a2*/*Nr4a1*, *Dusp1*, *Fos*/*Fosl2*, *Npas4*, *Per1*/*Per2*/*Per3*, *Foxo3*/*Foxo1*, and *Homer1*. A parallel downregulation of the NPAS4 protein interactor *Arnt* and its binding partner *Hif3a* mRNA were observed, as well as reduced transcript levels of downstream factors *Slc2a12* and *Rora* (which is responsible for ataxia and intellectual deficits [178,179]), contrasting with upregulation of the alternative interactor *Arnt2* mRNA [180,181,182,183,184]. With relevance to the osmotic homeostasis in ATM-null cerebellum, a strong downregulation was observed for *Dbp* as the transcription factor that controls the expression of alpha-fetoprotein and albumin (whose dysregulation is characteristic for A-T [4,5]), and is crucial for circadian regulation of synaptic plasticity [185,186,187]. The notion of changed nutrient and osmotic regulation was also supported by the downregulation of amino acid-sensing *Rragd* [104]. Downregulation was prominent for immune-regulating and damage-responsive protein kinase transcripts *Smg1*, *Sik1* and *Sgk1* [188,189,190], findings that also implicate altered RNA surveillance, osmotic and nutrient homeostasis in A-T pathology. 

A deficit in inflammatory responses was also evident from the downregulated transcripts of *Ccl27a*, *Sidt1*, *Il16*, *Rnf122* and *Serinc2* [191,192,193,194,195]. The deficiency of immunoglobulin/fibronectin-domain-containing *Boc* may contribute to the observed upregulation of many protocadherin, cadherin, and contactin pathway members (*Cdh6*, *Cdh9*, *Cdh10*, *Cdh19*, *Pcdh7*, *Pcdh10*, *Pcdh11x*, *Pcdh17*, *Pcdh18*, *Pcdh19*, *Cntn4*, *Cntn5* and *Cntnap5a*) [196]. 

In conclusion, pathway enrichment analyses of the transcriptome profile supported the novel concept that failure of ATM-mediated adaptation to osmotic/nutrient and perhaps oxidative stress, via altered USP2/ATMIN signals, leads to a generalised abnormality in neurotransmitter–neuropeptide signals from synaptic and dense core vesicles, with reduced immediate-early signals and impaired synaptic adhesion.

### 4.2. The Alternative Splice Profile of ATM-Null Mouse Cerebellum at 12 Months of Age

Previous studies of ATM dysfunction demonstrated its impact on alternative splicing [53], and of course the stimulus-/stress-dependent changes in phosphorylation cascades would first impact the splice apparatus before topoisomerase-dependent immediate-early reactions and more cumbersome chromatin unpackaging events permit the subsequent transcription adaptations. Overall, our interrogation of the transcriptome for factors with strong exon-splicing-index effects confirmed significant enrichments in three pathways. Neurotransmission (*Slc17a6*, *Slc18a2*, *Slc7a3*, *Slc5a7*, *Slco1a4*, *Scn3b*, *Kcnq5*, *Gabrq*, *Glra1*, *Glra3*, *Dbh*, *Wnk1*, *Car9*, *Micu3*), neuropeptides/neurotrophins (*Oprm1*, *Baiap3*, *Gnas*, *Dlk1*, *Dlg2*, *Rasgrf2*, *Ngef*, *Ecel1*, *Dgkk*, *Ccl27a*, *Pcsk5*, *Cpne6*, *Ptprd*, *Gfra1*) and synaptic adhesion (*Fxyd5*, *Cbln4*, *Cbln2*, *Nrip2*) modulation was prominent. *Baiap3* splice changes could contribute to impaired biogenesis of secretory vesicles, with consequences for the Ca^2+^ stimulated release of neurotransmitters and neuropeptides [197]. While cerebellar tissue has a broad expression profile, a neural cell culture model would express only a small subset of these molecules, so further validation experiments were focused on *Oprm1*, which exhibited an exceptional exon-splicing index of 39.7, and which acts to dampen glutamatergic neurotoxicity in the contacts between cerebellar granule neuron projections (parallel fibres) and Purkinje neuron dendrites [198,199,200], which is the cerebellar site most vulnerable to ataxia pathogenesis [201,202]. With a prominent negative exon-splicing index of −5.03 (*p* = 0.005), *Slc17a6* (encoding VGLUT2) also displayed evident adaptation of its exon structure, providing additional evidence that also glutamatergic climbing fibre-signalling is modulated by ATM. In contrast, *Slc17a7* (VGLUT1 in parallel fibres) showed a change only for its 3′-exon with nominal significance and an exon-splicing index of 1.6. While the ClariomD microarray has oligonucleotides to detect sequences within most exons, the Taqman RT-qPCR assays in contrast are optimised to detect exon–exon-boundaries, so a validation experiment by RT-qPCR can only confirm the dysregulation of a specific mRNA overall, and may detect whether it disappears for a specific exon, but will not quantify the selective inclusion/exclusion of an exon. Overall, it is important to be cautious regarding the value of this splicing profile, because dysregulations of a complete mRNA may not be equally represented by every oligonucleotide, mimicking true alternative splice changes, and because experimental quantitative validation across species is cumbersome. Thus, we consider these data as preliminary screen.

### 4.3. Differential Detergent Fractionation of Adult Mouse Cerebellum and SH-SY5Y Neuroblastoma Cells Detects ATM Mainly in Cytosol

For validation of these mouse findings in the human species, a knockdown of ATM in neuroblastoma SH-SY5Y cells was employed, taking into account the previous usefulness of such human in vitro modelling projects in autophagy and chemoresistance studies of ATM [203,204]. Neuroblastoma cell lines are known to represent a mixed population, termed N-type (neural) and S-type (substrate-adherent, epithelial-like) cells. While N-type cells are neuroblast-like with little cytoplasm and few neuritic processes, the S-type cells have a bigger cytoplasm and flattened morphology with strong attachment to the substrate [205,206,207,208]. Unexpectedly, *ATM*-KD SH-SY5Y cells displayed gross alterations in appearance, with larger and flattened cell bodies without processes, while the non-targeting control (NT CTRL) cells retained an overall neuroblast-like appearance and displayed short neuritic processes (Figure S2). Thus, flattened cells without processes that are predominant for *ATM*-KD cells could reflect a shift in neuroblastoma cell populations towards S-type cells. The remodelling of Ca^2+^ signalling was already demonstrated to be altered in S-type cells [206]. Given that SH-SY5Y cells were already shown to require ATM mediated phosphorylation of CREB protein at serine 133, to enable retinoic acid induced differentiation [209], this change of gross morphology in *ATM*-KD cells may represent a loss of differentiation. Indeed, dysregulation of retinoic acid dependent differentiation regulators was also evident in the 12-month-old ATM-null mouse cerebellar transcriptome, where most components of this well characterised pathway [210,211,212] were strongly dysregulated, displaying increased *Nrip2* (Figure 2a), *Nrip3*, *Rorb*, *Creb5*, *Crebl2* and *Zfhx3* mRNA levels versus decreased expression of *Tcf4*, *Crtc2*, *Foxo3* and the ataxia disease gene *Rora*, as well as downstream *Itpr1* [213,214]. A more proliferative state of the *ATM*-KD cells may result in reduced expression of many neurotransmission factors, potentially explaining the drastic downregulation of *OPRM1* in *ATM*-KD neuroblastoma cells, as opposed to increased *Oprm1* in the old ATM-null cerebellum (Figure 6d). Although some dysregulations of neuropeptide signalling were massive in the old ATM-null mouse cerebellum and were very relevant for phenotypes of A-T—e.g., the increases in mRNA of growth hormone inhibitor somatostatin (*Sst*), vasodilator preprotachykinin (*Tac1*) and the tachykinin receptor (*Tacr1*), as well as the glutamate-excitability factor VGLUT2 (encoded by *Slc17a6*)—the SH-SY5Y neuroblastoma line did not express these genes. It is also important to consider that glutamate availability is very restricted for excitable neurons in vivo, but provided constantly in overdose to cultured neuroblastoma cells. Thus, our in vitro model had very limited value to model and study the ATM-dependent stress-adaptations of neurotransmitter- and neuropeptide-containing vesicles, but seemed quite helpful for the study of upstream factors in the interactome of ATM and of immediate-early responses in the nucleus. 

The use of differential detergents to achieve subcellular fractionation of the old adult mouse cerebellum and of SH-SY5Y neuroblastoma line, localised ATM mainly to the cytosol, both in unstressed and in stressed conditions, in WT cells and *ATM*-KD cells (Figure 3, Figure S3). The absence of ATM from the nucleus is in excellent agreement with subsequent findings that characteristic ATM-null cerebellar transcriptome profile anomalies such as *USP2* and *PER1* downregulations could not be elicited robustly by the DNA DSB stressor bleomycin in vitro, but were mirrored best by osmotic stress instead (Figure 4). Our observations that ATM is almost exclusively found within cytoplasmic fractions of the cerebellum and SH-SY5Y cells are in excellent agreement with a previous immunohistochemical study that localised ATM in the cytosol of cerebellar tissue Purkinje neurons from a mouse mutant [32], but they contrast with human reports and with the immunohistochemical observation of ATM in the nucleus of cerebellar Purkinje and granule neurons, once they are dissected and kept in organotypic slice cultures [215,216]. While nuclear ATM clearly has a role for DNA repair in proliferating cells, these fractionation findings emphasise the urgent need to understand what the functions of cytosolic ATM in postmitotic neurons are, and how impaired stress adaptation there might trigger a neurodegenerative process. Given the inefficiency of ATM kinase inhibition by KU-55933 to reproduce transcript changes observed in cerebellar tissue and upon *ATM*-KD in vitro, we consider the possibility that cytoplasmic ATM acts as a protein scaffold and interaction platform, rather than a kinase in cytoplasmic signalling. This was already proposed when cytoplasmic ATM was demonstrated to serve as a docking site for PP2A to dephosphorylate AKT and thereby regulate cell death upon ER-stress via an ATM-AKT-GSK3β-αNAC/γTX signalling axis [217]. Further research is necessary to elucidate the potential functions of ATM as a protein scaffold in the cytoplasm.

### 4.4. Validation Work in SH-SY5Y Cells Shows ATM-Deficiency to Impair the CQ-Triggered Regulation of Postsynaptic Calcium Release Channel ITPR1, in Parallel to Immediate-Early Transcripts PER1/NR4A1

The mechanistic validation experiments in this study (Figure 4, Figure 5 and Figure S4) focused on A-T-phenotype-related factors, documenting consistent reductions for ITPR1 levels upon ATM deficiency. Given that ATM is a member of the phosphatidylinositol 3′ kinase-like kinase (PIKK) enzyme family, it may have functional interaction with the inositol-1,4,5-trisphosphate-receptor IP3R, so this decrease of *ITPR1* mRNA and IP3R abundance may also represent a crucial primary loss-of-function event in autosomal recessive A-T. Genetic loss-of-function of IP3R has a profound impact on the calcium-dependent excitability of Purkinje neurons and was repeatedly observed as sufficient to cause hereditary progressive cerebellar neurodegeneration, with deletion of one *ITPR1* gene copy via haploinsufficiency triggering ataxia inheritance in autosomal dominant manner [218,219]. Thus, our observation of IP3R abundance reduction below 50% in *ATM*-KD neuroblastoma cells (Figure 5b) emerges from the transcriptome profile validation work as arguably the most important molecular event, which might explain the preferential affection of cerebellar neurons [57]. In this context, it is important to note that parallel loss of IP3R together with two more ataxia-responsible proteins was previously demonstrated in cerebellar tissue of A-T patients [66], namely the calcium homeostasis factor INPP5A (also known as Type I Inositol 1,4,5-Trisphosphate 5-Phosphatase) [220], and CA8 (also known as Carbonic Anhydrase 8) [221,222]. Furthermore, a protein complex of ITPR1 as endoplasmic reticulum Ca^2+^ homeostasis regulator with the mitochondrial HSP70-family member/chaperone GRP75 (=HSPA9) and the mitochondrial voltage-gated Ca^2+^ homeostasis channel VDAC1 was previously shown to mediate ATM dysfunction in bronchial cells after nutrient stress [30]. A very similar expression alteration in the ATM-null mouse cerebellar transcriptome may therefore be relevant, where downregulation of the ataxia gene *Itpr1* occurred in parallel with downregulation of the HSP70-family member/chaperone *Hspa12a* and several voltage-gated Ca^2+^ homeostasis channels in the plasma membrane (*Cacna1a*, *Cacna1c*, *Cacna1d*, *Cacna1g*). Cytosolic HSPA12A protein has a very specific role, modulating its interactor SORL1 with downstream GFRA1/2 [223,224], all of which showed downregulated mRNA levels in our ATM-null mouse cerebellar transcriptome profiling study, suggesting that the sorting of trophic signalling receptors is abnormally regulated. In addition, the receptor tyrosine kinase ERBB2 is regulated both by SORL1 and by USP2 [225,226], and this may underlie the reduced cerebellar levels of *Erbb2ip* as a factor responsible for the surface localisation of glutamate receptors [227]. In analogy to the impact of nutrient deficits as stressors via ATM on endoplasmic reticulum and mitochondria homeostasis as previously published [30], the transcriptional dysregulation of ITPR1, HSPA12A (instead of HSPA9/GRP75) and its interactors SORL1 and ERBB2 in ATM-null cerebellum may therefore constitute the primary pathogenesis pathway and explain why, when neurotransmitter receptors/transporters/neuropeptide modulators are not in the right position in polarised processes, trophic signalling deficits ensue, and tissue shrinkage ensues over time. Given that SORL1, GFRA1/2, ERBB2, are poorly or not expressed in SH-SY5Y neuroblastoma cells according to the Human Protein Atlas (last accessed on 12 September 2023), we made no attempt to model this trophic pathogenesis cascade in vitro.

The reduction of IP3R would also mediate a postsynaptic excitability deficit, and contribute to the diminished transcriptional response of immediate-early transcripts such as *PER1* and *NR4A1*.

Overall, these molecular insights are well compatible with previous reports in ATM-deficient cultured hippocampal slices and primary cortical neurons, where presynaptic pathology was identified as a primary problem [38], and where an increase of both, excitatory and inhibitory signalling was documented electrophysiologically [228,229].

## 5. Conclusions

Overall, our genome-wide RNA profile provided useful knowledge to identify factors that might underlie the growth deficit (somatostatin and neuropeptide-Y) and vasodilatation phenotypes (tachykinins and *Ecel1*) of A-T, and to define the mechanistic overlap of A-T with the *Itpr1*–triggered monogenic variants of cerebellar ataxia. The data in this project suggested that the presence of cytosolic ATM in postmitotic cerebellar neurons serves as an important modulator of the transcriptional regulation of excitability factors in response to ageing, and to osmotic stress more than nutrient or oxidative stress. Validations in neuroblastoma culture could largely reproduce crucial insights and the prominent alterations found in the cerebellar transcriptome: Strong reduction of *Atm* levels was reflected in similar strong decreases of its interactor *Usp2*, the mainly Purkinje-neuron-expressed Ca^2+^-excitability modulator *Itpr1* mRNAs, and immediate-early signalling factors such as *Per1* and *Nr4a1*. Future experiments have to determine to what degree a ubiquitous or selective affection of transcripts in the well-known cerebellar cell types and circuitry pathways exists, as shown in the Graphical Abstract (which contains a scheme from [230] with modifications).

## Figures and Tables

**Table 1:** Consistently dysregulated factors where ATM-null mouse cerebellar transcriptome data agreed with published A-T patient cerebellar proteome.

ID	Cerebellum 12 Months Avg (log2)		Fold Change	FDR *p*-Val	Gene Symbol
	KO	WT			
TC1100002122.mm.1	10.58	9.71	1.83	0.0081	Nefh
TC1400000965.mm.1	13.04	12.06	1.98	0.0101	Nefl
TC0300001124.mm.1	11.24	10.59	1.57	0.0133	Gstm5
TC0200003051.mm.1	11.62	11.12	1.41	0.0145	Pip4k2a
TC1500001133.mm.1	8.34	7.02	2.51	0.0165	Pde1b
TC1700002629.mm.1	10.81	10.14	1.59	0.0168	Cox7a2l
TC0800001522.mm.1	10.82	9.81	2.01	0.0176	Tubb3
TC1700002597.mm.1	9.83	9.41	1.34	0.0182	Slc8a1
TC0900001605.mm.1	13.96	13.37	1.51	0.0234	Rpl14
TC1100002119.mm.1	10.38	9.67	1.64	0.0238	Uqcr10
TC0400001770.mm.1	6.92	6.39	1.45	0.0249	Padi2
TC0500000640.mm.1	10.76	9.47	2.44	0.0249	Uchl1
TC1100000302.mm.1	13.33	12.97	1.29	0.0274	Rtn4
TC1800001198.mm.1	10.35	9.74	1.53	0.029	Ndufa2
TC1200000990.mm.1	11.21	10.81	1.32	0.0297	Psmc1
TC1100002486.mm.1	13.52	13.03	1.4	0.0303	Npm1
TC0800002382.mm.1	7.67	7.12	1.46	0.0307	Ncan
TC0600001689.mm.1	9.15	8.64	1.42	0.031	Cmas
TC0100000551.mm.1	12.24	11.8	1.36	0.0321	Map2
TC0100000519.mm.1	11.71	11.35	1.29	0.0325	Eef1b2
TC0500001724.mm.1	7.45	6.74	1.64	0.0327	Fscn1
TC1000001298.mm.1	12.64	12.15	1.4	0.0348	Nap1l1
TC1400000485.mm.1	12.45	12.12	1.25	0.0351	Psmc6
TC0X00001071.mm.1	12.79	12.39	1.33	0.0362	Pgk1
TC1600001590.mm.1	12.29	12.81	−1.43	0.0365	Stxbp5l
TC1000000571.mm.1	10.77	10.32	1.37	0.04	Ppa1
TC1600000750.mm.1	12.72	12.34	1.3	0.0408	Tomm70a
TC1300001779.mm.1	13.02	12.04	1.98	0.0413	Tubb2a
TC1000001684.mm.1	13.14	13.88	−1.67	0.0447	Syne1
TC1700001012.mm.1	10.78	9.87	1.87	0.0447	Rpl36
TC1200001971.mm.1	14.08	13.67	1.33	0.0449	Atp6v1d
TC0900000705.mm.1	13.46	13.07	1.31	0.0458	Cox5a
TC0600001931.mm.1	12.22	12.71	−1.41	0.0474	Cadps2
TC0700000832.mm.1	11.29	12.09	−1.74	0.0481	Slc17a7
TC0700001988.mm.1	8.65	9.08	−1.34	0.0481	Dpysl4
TC1200001568.mm.1	13.51	13.15	1.28	0.05	Rps7

## Data Availability

The complete gene expression data set was deposited publicly in the Gene Expression Omnibus under accession number GSE241955.

## Supplementary Materials

The following supporting information can be downloaded at: https://www.mdpi.com/article/10.3390/cells12192399/s1, Figure S1: RT-qPCR validation of cerebellar transcript dysregulations in 1.5–3-month-old ATM-null mice shows early onset of immediate-early mRNA reductions, suggesting insufficient excitability; Figure S2: Modelling stable *ATM*-deficiency in vitro via knockdown in SH-SY5Y neuroblastoma cells; Figure S3: Subcellular localisation of ATM to SH-SY5Y cytoplasm is unchanged after CQ and BLEO stress; Figure S4: RT-qPCR analysis of the modelling of cerebellar dysregulations in SH-SY5Y cells, testing different stressors, as well as ATM deficiency versus kinase inhibition; Table S1: The global transcriptome profile of the ATM-null cerebellum.

## Abbreviations

ACTB	β-Actin
AFP	Alpha-Fetoprotein
AKT	AKT serine/threonine kinase
AMPA	α-amino-3-hydroxy-5-methyl-4-isoxazolepropionic acid
ANOVA	Analysis of variance
AOA2	Ataxia with oculomotor apraxia type 2
AP1B1	Adaptor Related Protein Complex 1 Subunit Beta 1
AP2B1	Adaptor Related Protein Complex 2 Subunit Beta 1
ARNT2	Aryl Hydrocarbon Receptor Nuclear Translocator 2
ASTN2	Astrotactin 2
A-T	Ataxia Telangiectasia
ATM	Ataxia Telangiectasia Mutated
ATMIN	ATM Interactor
ATP2B2	ATPase Plasma Membrane Ca2+ Transporting 2
ATP2B4	ATPase Plasma Membrane Ca2+ Transporting 4
ATR	Ataxia Telangiectasia And Rad3-Related Protein
ATXN2	Ataxin 2
BAIAP3	BAI1 Associated Protein 3
BCA	Bicinchoninic acid
beta-NAP	Neuronal Adaptin-like beta-subunit Protein
BLEO	Bleomycin
BMP1	Bone Morphogenetic Protein 1
BMP2	Bone Morphogenetic Protein 2
BMP7	Bone Morphogenetic Protein 7
BMPR1B	Bone Morphogenetic Protein Receptor Type 1B
BMT	Bone Marrow transplantation
BSA	Bovine Serum Albumin
C4B	Complement Component 4B
CA8	Carbonic Anhydrase 8
CACNA1A	Calcium Voltage-Gated Channel Subunit Alpha1 A
CACNA1C	Calcium Voltage-Gated Channel Subunit Alpha1 C
CACNA1D	Calcium Voltage-Gated Channel Subunit Alpha1 D
CACNA1G	Calcium Voltage-Gated Channel Subunit Alpha1 G
CADPS2	Calcium Dependent Secretion Activator 2CAMK2A
CAMK4	Calcium/Calmodulin Dependent Protein Kinase IV
Car9	murine Carbonic Anhydrase 9
CBLN2	Cerebellin 2 Precursor
CBLN4	Cerebellin 4 Precursor
CCL27A	C-C Motif Chemokine Ligand 27
CCNL2	Cyclin L2
CDH10	Cadherin 10
CDH19	Cadherin 19
CDH6	Cadherin 6
CDH9	Cadherin 9
cDNA	complementary DNA
CEB	cytosolic extract buffer
CHK2	Checkpoint Kinase 2
CHKA	Choline Kinase Alpha
CHRM2	Cholinergic Receptor Muscarinic 2
CHRM3	Cholinergic Receptor Muscarinic 3
CHRM5	Cholinergic Receptor Muscarinic 5
CHRNA4	Cholinergic Receptor Nicotinic Alpha 4 Subunit
CHRNA6	Cholinergic Receptor Nicotinic Alpha 6 Subunit
CHRNA7	Cholinergic Receptor Nicotinic Alpha 7 Subunit
CHRNB3	Cholinergic Receptor Nicotinic Beta 3 Subunit
CHRNB4	Cholinergic Receptor Nicotinic Beta 4 Subunit
CNR1	Cannabinoid Receptor 1
CNTN4	Contactin 4
CNTN5	Contactin 5
CNTN6	Contactin 6
CNTNAP5A	Contactin Associated Protein Family Member 5
CPNE6	Copine 6
CQ	Chloroquine
CREB5	CAMP Responsive Element Binding Protein 5
CREBL2	CAMP Responsive Element Binding Protein Like 2
CRTC2	CREB Regulated Transcription Coactivator 2
CRY1	Cryptochrome Circadian Regulator 1
CRY2	Cryptochrome Circadian Regulator 2
CSF1R	Colony Stimulating Factor 1 Receptor
CYP46A1	Cytochrome P450 Family 46 Subfamily A Member 1
DBH	Dopamine Beta-Hydroxylase
DBP	D-Box Binding PAR BZIP Transcription Factor
DDR	DNA Damage Response
DGKK	Diacylglycerol Kinase Kappa
DLG2	Discs Large MAGUK Scaffold Protein 2
DLK1	Delta Like Non-Canonical Notch Ligand 1
DMSO	Dimethyl sulfoxide
DNA	Deoxyribonucleic acid
DOCK10	Dedicator Of Cytokinesis 10
DRD2	Dopamine Receptor D2
DRD5	Dopamine Receptor D5
DSB	Double-Strand Breaks
DUSP1	Dual Specificity Phosphatase 1
EBF3	EBF Transcription Factor 3
ECEL1	Endothelin Converting Enzyme Like 1
EFNA2	Ephrin A2
EFNA3	Ephrin A3
EFNA5	Ephrin A5
EFNB3	Ephrin B3
EPHA3	EPH Receptor A3
EPHA4	EPH Receptor A4
EPHA5	EPH Receptor A5
ERBB2	Erb-B2 Receptor Tyrosine Kinase 2
ERBB3	Erb-B2 Receptor Tyrosine Kinase 3
ERBB4	Erb-B2 Receptor Tyrosine Kinase 4
FAT2	FAT Atypical Cadherin 2
FATC	FRAP, ATM, TRRAP C-terminal
FCS	Fetal Calf Serum
FDR	False Discovery Rate
FGF13	Fibroblast Growth Factor 13
FGF14	Fibroblast Growth Factor 14
FGF18	Fibroblast Growth Factor 18
FGF5	Fibroblast Growth Factor 5
FGFR1OP2	FGFR1 Oncogene Partner 2
FGFR3	Fibroblast Growth Factor Receptor 3
FOS	Fos Proto-Oncogene
FOSL2	FOS Like 2
FOXO1	Forkhead Box O1
FOXO3	Forkhead Box O3
FRDA	Friedreich’s Ataxia
FSCN1	Fascin Actin-Bundling Protein 1
FXYD5	FXYD Domain Containing Ion Transport Regulator 5
FZD4	Frizzled Class Receptor 4
FZD8	Frizzled Class Receptor 8
GABA	Gamma-aminobutyric acid
GABARAPL1	GABA Type A Receptor Associated Protein Like 1
GABRA2	Gamma-Aminobutyric Acid Type A Receptor Subunit Alpha2
GABRA3	Gamma-Aminobutyric Acid Type A Receptor Subunit Alpha3
GABRA5	Gamma-Aminobutyric Acid Type A Receptor Subunit Alpha5
GABRA6	Gamma-Aminobutyric Acid Type A Receptor Subunit Alpha6
GABRB1	Gamma-Aminobutyric Acid Type A Receptor Subunit Beta1
GABRE	Gamma-Aminobutyric Acid Type A Receptor Subunit Epsilon
GABRG1	Gamma-Aminobutyric Acid Type A Receptor Subunit Gamma1
GABRG2	Gamma-Aminobutyric Acid Type A Receptor Subunit Gamma2
GABRG3	Gamma-Aminobutyric Acid Type A Receptor Subunit Gamma3
GABRQ	Gamma-Aminobutyric Acid Type A Receptor Subunit Theta
GAPDH	Glyceraldehyde-3-Phosphate Dehydrogenase
GBA2	Glucosylceramidase Beta 2
GFRA1	GDNF Family Receptor Alpha 1
GFRA2	GDNF Family Receptor Alpha 2
GH	growth hormone
GLRA1	Glycine Receptor Alpha 1
GLRA2	Glycine Receptor Alpha 2
GLRA3	Glycine Receptor Alpha 3
GLRA4	Glycine Receptor Alpha 4
GNAS	GNAS Complex Locus
GO	Gene Ontology
GPR165	G Protein-Coupled Receptor 165
GRID1	Glutamate Ionotropic Receptor Delta Type Subunit 1
GRID2	Glutamate Ionotropic Receptor Delta Type Subunit 2
GRID2IP	Grid2 Interacting Protein
GRIK2	Glutamate Ionotropic Receptor Kainate Type Subunit 2
GRIN2B	Glutamate Ionotropic Receptor NMDA Type Subunit 2B
GRIN2C	Glutamate Ionotropic Receptor NMDA Type Subunit 2C
GRIN3A	Glutamate Ionotropic Receptor NMDA Type Subunit 3A
GRIP1	Glutamate Receptor Interacting Protein 1
GRM3	Glutamate Metabotropic Receptor 3
GRM4	Glutamate Metabotropic Receptor 4
GRM5	Glutamate Metabotropic Receptor 5
GRM8	Glutamate Metabotropic Receptor 8
GRP75/HSPA9	Heat Shock Protein Family A (Hsp70) Member 9
GSK3β	Glycogen Synthase Kinase 3 Beta
H_2_O_2_	Hydrogen peroxide
HDMX	Human ortholog of mouse MDMX (also known as MDM4)
HIF3A	Hypoxia Inducible Factor 3 Subunit Alpha
HOMER1	Homer Scaffold Protein 1
HSP60	60 KDa Heat Shock Protein, Mitochondrial
HSP70	Heat Shock 70 KDa Protein 4
HSPA12A	Heat Shock Protein Family A (Hsp70) Member 12A
IGF1	Insulin Like Growth Factor 1
IGF2	Insulin Like Growth Factor 2
IGF2R	Insulin Like Growth Factor 2 Receptor
IGFLR1	IGF Like Family Receptor 1
IgG	Immunoglobulin G
IL16	Interleukin 16
IL18	Interleukin 18
IL20RB	Interleukin 20 Receptor Subunit Beta
IL33	Interleukin 33
IL34	Interleukin 34
INPP5A	Type I Inositol 1,4,5-Trisphosphate 5-Phosphatase
ITPR1/IP3R1	Inositol 1,4,5-Trisphosphate Receptor Type 1
JUN	Jun Proto-Oncogene
KAT5	Lysine Acetyltransferase 5
KCNQ5	Potassium Voltage-Gated Channel Subfamily Q Member 5
KD	knockdown
KDR	Kinase Insert Domain Receptor
KIT	KIT Proto-Oncogene
KO	knockout
KU	KU-55933
LCDV	Large Dense Core Vesicles
LIFR	LIF Receptor Subunit Alpha
LY	LY-294002
MAPK9/JNK2	Mitogen-Activated Protein Kinase 9
MDM4	MDM4 Regulator Of P53
MICU3	Mitochondrial Calcium Uptake Family Member 3
miR	microRNA
MLB	mitochondrial lysis buffer
MME	Membrane Metalloendopeptidase
MPP4	MAGUK P55 Scaffold Protein 4
MRE11	Double-Strand Break Repair Protein MRE11
MRN	MRE11-RAD50-NBS1
mRNA	messenger RNA
NaARS	Sodium Arsenite
NBS1	Nibrin
NECAB1	N-Terminal EF-Hand Calcium Binding Protein 1
NEFL	Neurofilament Light Chain
NEFM	Neurofilament Medium Chain
ng	nanogram
NGEF	Neuronal Guanine Nucleotide Exchange Factor
NGFRAP1	Nerve Growth Factor Receptor Associated Protein 1
NPAS4	Neuronal PAS Domain Protein 4
NPY	Neuropeptide Y
NPY1R	Neuropeptide Y Receptor Y1
NPY4R	Neuropeptide Y Receptor Y4
NR4A1	Nuclear Receptor Subfamily 4 Group A Member 1
NR4A2	Nuclear Receptor Subfamily 4 Group A Member 2
NR4A3	Nuclear Receptor Subfamily 4 Group A Member 3
NRG1	Neuregulin 1
NRG3	Neuregulin 3
NRIP2	Nuclear Receptor Interacting Protein 2
NRIP3	Nuclear Receptor Interacting Protein 3
NSG2	Neuronal Vesicle Trafficking Associated 2
NT CTRL	Non-targeting control shRNA
NTF3	Neurotrophin 3
NTRK3	Neurotrophic Receptor Tyrosine Kinase 3
NTS	Neurotensin
NTSR1	Neurotensin Receptor 1
NTSR2	Neurotensin Receptor 2
OMD	Osteomodulin
OPRK1	Opioid Receptor Kappa 1
OPRL1	Opioid Related Nociceptin Receptor 1
OPRM1	Opioid Receptor Mu 1
PBS	Phosphate Buffered Saline
PCDH10	Protocadherin 10
PCDH11X	Protocadherin 11 X-Linked
PCDH17	Protocadherin 17
PCDH18	Protocadherin 18
PCDH19	Protocadherin 19
PCDH7	Protocadherin 7
PCSK1	Proprotein Convertase Subtilisin/Kexin Type 1
PCSK1N	Proprotein Convertase Subtilisin/Kexin Type 1 Inhibitor
PCSK5	Proprotein Convertase Subtilisin/Kexin Type 5
PDGFC	Platelet Derived Growth Factor C
PDGFRA	Platelet Derived Growth Factor Receptor Alpha
PDGFRL	Platelet Derived Growth Factor Receptor Like
PER1	Period Circadian Regulator 1
PER2	Period Circadian Regulator 2
PER3	Period Circadian Regulator 3
PI3K	Phosphoinositide 3-kinase
PIKK	Phosphoinositide 3-kinase-related kinases
PMSF	Phenylmethylsulfonyl fluoride
PP2A	Protein phosphatase 2A
PPP2R2B	Protein Phosphatase 2 Regulatory Subunit Bbeta
PTPRD	Protein Tyrosine Phosphatase Receptor Type D
QPCT	Glutaminyl-Peptide Cyclotransferase
RAD50	RAD50 Double Strand Break Repair Protein
RASGRF2	Ras Protein Specific Guanine Nucleotide Releasing Factor 2
RELN	Reelin
RESP18	Regulated Endocrine Specific Protein 18
RGS4	Regulator Of G Protein Signaling 4
RIN	RNA integrity number
RNA	Ribonucleic acid
RNF122	Ring Finger Protein 122
RORA	RAR Related Orphan Receptor A
RORB	RAR Related Orphan Receptor B
RRAGD	Ras Related GTP Binding D
RTN4	Reticulon 4
RT-qPCR	Reverse transcription-quantitative polymerase chain reaction
SAM68	Src-Associated In Mitosis 68 KDa Protein
SCN3B	Sodium Voltage-Gated Channel Beta Subunit 3
SDS-PAGE	Sodium dodecyl-sulfate polyacrylamide gel electrophoresis
SEM	Standard error of the mean
SEPT9	Septin 9
SERINC2	Serine Incorporator 2
SFRP1	Secreted Frizzled Related Protein 1
SFRP5	Secreted Frizzled Related Protein 5
SGK1	Serum/Glucocorticoid Regulated Kinase 1
shATM	shRNA targeting *ATM*
shRNA	Short hairpin RNA
SIDT1	SID1 Transmembrane Family Member 1
SIK1	Salt Inducible Kinase 1
SLC17A6/VGLUT 2	Solute Carrier Family 17 Member 6
SLC17A7/VGLUT1	Solute Carrier Family 17 Member 7
SLC18A2	Solute Carrier Family 18 Member A2
SLC1A1	Solute Carrier Family 1 Member 1
SLC1A2	Solute Carrier Family 1 Member 2
SLC1A3	Solute Carrier Family 1 Member 3
SLC1A4	Solute Carrier Family 1 Member 4
SLC1A6	Solute Carrier Family 1 Member 6
SLC25A22	Solute Carrier Family 25 Member 22
SLC2A12	Solute Carrier Family 2 Member 12
SLC32A1	Solute Carrier Family 32 Member 1
SLC5A7	Solute Carrier Family 5 Member 7
SLC6A11	Solute Carrier Family 6 Member 11
SLC7A3	Solute Carrier Family 7 Member 3
SLCO1A4	Solute Carrier Organic Anion Transporter Family Member 1A2
SLITRK3	SLIT And NTRK Like Family Member 3
SLITRK5	SLIT And NTRK Like Family Member 5
SLITRK6	SLIT And NTRK Like Family Member 6
SMG1	SMG1 Nonsense Mediated MRNA Decay Associated PI3K Related Kinase
SOCS7	Suppressor Of Cytokine Signaling 7
SORCS1	Sortilin Related VPS10 Domain Containing Receptor 1
SORCS2	Sortilin Related VPS10 Domain Containing Receptor 2
SORL1	Sortilin Related Receptor 1
SOX10	SRY-Box Transcription Factor 10
ss-cDNA	single-stranded cDNA
SST	Somatostatin
SSTR1	Somatostatin Receptor 1
SSTR2	Somatostatin Receptor 2
SVBP	Small Vasohibin Binding Protein
SYNE1	Spectrin Repeat Containing Nuclear Envelope Protein 1
TAC	Transcriptome Analysis Console
TAC1	Tachykinin Precursor 1
TACR1	Tachykinin Receptor 1
TACR3	Tachykinin Receptor 3
TBP	TATA-Box Binding Protein
TBS	Tris Buffered Saline
TBS-T	TBS with 0.1% Tween-20 detergent
TCF4	Transcription Factor 4
TGFBR1	Transforming Growth Factor Beta Receptor 1
TGFBR2	Transforming Growth Factor Beta Receptor 2
TNFRSF13C	TNF Receptor Superfamily Member 13C
TNFRSF21	TNF Receptor Superfamily Member 21
TOP1cc	Topoisomerase-1 cleavage complexes
TP53	Tumor Protein P53
tRNA	transfer RNA
TUBA	α-tubulin
USP2	Ubiquitin Specific Peptidase 2
UVB	Ultraviolet-B
V(D)J	Variability–Diversity–Joining Rearrangement
VAMP1	Vesicle Associated Membrane Protein 1
VCL	Vinculin
VDAC1	Voltage Dependent Anion Channel 1
WNK1	WNK Lysine Deficient Protein Kinase 1
WNT3	Wnt Family Member 3
WNT7A	Wnt Family Member 7A
WT	Wildtype
ZFHX3	Zinc Finger Homeobox 3
αNAC	Nascent Polypeptide Associated Complex Subunit Alpha
γTX	γ-taxilin
μg	microgram
µm	micrometer
